# ResNetKhib: a novel cell type-specific tool for predicting lysine 2-hydroxyisobutylation sites via transfer learning

**DOI:** 10.1093/bib/bbad063

**Published:** 2023-03-04

**Authors:** Xiaoti Jia, Pei Zhao, Fuyi Li, Zhaohui Qin, Haoran Ren, Junzhou Li, Chunbo Miao, Quanzhi Zhao, Tatsuya Akutsu, Gensheng Dou, Zhen Chen, Jiangning Song

**Affiliations:** Collaborative Innovation Center of Henan Grain Crops, and Key Laboratory of Rice Biology in Henan Province, College of Agronomy, Henan Agricultural University, China; State Key Laboratory of Cotton Biology, Institute of Cotton Research of Chinese Academy of Agricultural Sciences, Anyang 455 000, Henan, China; Zhengzhou Research Base, State Key Laboratory of Cotton Biology, School of Agricultural Sciences, Zhengzhou University, Zhengzhou, China; Monash University, Australia. He is currently a professor at the College of Information Engineering, Northwest A&F University, China; Collaborative Innovation Center of Henan Grain Crops, and Key Laboratory of Rice Biology in Henan Province, College of Agronomy, Henan Agricultural University, China; Collaborative Innovation Center of Henan Grain Crops, and Key Laboratory of Rice Biology in Henan Province, College of Agronomy, Henan Agricultural University, China; Collaborative Innovation Center of Henan Grain Crops, and Key Laboratory of Rice Biology in Henan Province, College of Agronomy, Henan Agricultural University, China; Collaborative Innovation Center of Henan Grain Crops, and Key Laboratory of Rice Biology in Henan Province, College of Agronomy, Henan Agricultural University, China; Collaborative Innovation Center of Henan Grain Crops, College of Agronomy, and Key Laboratory of Rice Biology in Henan Province, Henan Agricultural University, China; DEng degree in information engineering in 1989 from the University of Tokyo, Japan. Since 2001, he has been a professor in the Bioinformatics Center, Institute for Chemical Research, Kyoto University, Japan; College of Agronomy, Henan Agricultural University; Collaborative Innovation Center of Henan Grain Crops, Key Laboratory of Rice Biology in Henan Province and Center for Crop Genome Engineering, College of Agronomy, Henan Agricultural University, China; Monash Biomedicine Discovery Institute, Monash University

**Keywords:** transfer learning, sequence analysis, prediction, lysine 2-hydroxyisobutyrylation, protein post-translational modification, deep learning

## Abstract

Lysine 2-hydroxyisobutylation (K_hib_), which was first reported in 2014, has been shown to play vital roles in a myriad of biological processes including gene transcription, regulation of chromatin functions, purine metabolism, pentose phosphate pathway and glycolysis/gluconeogenesis. Identification of K_hib_ sites in protein substrates represents an initial but crucial step in elucidating the molecular mechanisms underlying protein 2-hydroxyisobutylation. Experimental identification of K_hib_ sites mainly depends on the combination of liquid chromatography and mass spectrometry. However, experimental approaches for identifying K_hib_ sites are often time-consuming and expensive compared with computational approaches. Previous studies have shown that K_hib_ sites may have distinct characteristics for different cell types of the same species. Several tools have been developed to identify K_hib_ sites, which exhibit high diversity in their algorithms, encoding schemes and feature selection techniques. However, to date, there are no tools designed for predicting cell type-specific K_hib_ sites. Therefore, it is highly desirable to develop an effective predictor for cell type-specific K_hib_ site prediction. Inspired by the residual connection of ResNet, we develop a deep learning-based approach, termed ResNetKhib, which leverages both the one-dimensional convolution and transfer learning to enable and improve the prediction of cell type-specific 2-hydroxyisobutylation sites. ResNetKhib is capable of predicting K_hib_ sites for four human cell types, mouse liver cell and three rice cell types. Its performance is benchmarked against the commonly used random forest (RF) predictor on both 10-fold cross-validation and independent tests. The results show that ResNetKhib achieves the area under the receiver operating characteristic curve values ranging from 0.807 to 0.901, depending on the cell type and species, which performs better than RF-based predictors and other currently available K_hib_ site prediction tools. We also implement an online web server of the proposed ResNetKhib algorithm together with all the curated datasets and trained model for the wider research community to use, which is publicly accessible at https://resnetkhib.erc.monash.edu/.

## Introduction

As one of the most important reversible protein post-translational modifications (PTMs), lysine 2-hydroxyisobutylation (K_hib_) was initially discovered on histones in male germ cells by Dai *et al*. in 2014 [1]. K_hib_ introduces a steric bulk with a mass shift of +86.03 Da and neutralizes the positive charge of the lysine [1, 2]. As an evolutionarily conserved and widely distributed PTM, K_hib_ has been identified in both eukaryotes and prokaryotes cells [1, 3], and was reported to share acyltransferases and deacylated with the widely studied lysine acetylation [4]. Histone 2-hydroxyisobutylation is associated with active gene transcription in spermatogenic cells and involved in the expression of disease resistance genes [4, 5], while 2-hydroxyisobutylation modification in non-histones is related to a variety of energy conversion processes, including tricarboxylic acid cycle, glycolysis, gluconeogenesis, etc. [4].

In order to fully understand the biological functions and processes roles of K_hib_-modified proteins and the corresponding K_hib_ sites, large-scale proteomic analyses of 2-hydroxyisobutylation have been carried out recently. Wu *et al*. [4] deeply studied the K_hib_ sites on histones and non-histones upon suberoylanilide hydroxamic acid (SAHA) treatment and found 8765 K_hib_ sites on 2484 mammalian proteins. It was found that K_hib_ proteins participated in the function of the ribosome, glycolysis/gluconeogenesis and transcription. Huang *et al*. [6] reported the first global proteomic profiling of K_hib_ substrates in human cells and discovered both the ‘writers’ and ‘erasers’ for histone K_hib_ marks. Zhang *et al*. [7] revealed the alteration in the actin cytoskeleton pathway of K_hib_ protein in oral squamous cell carcinoma through liquid chromatography and mass spectrometry/mass spectrometry (LC–MS/MS)-based modified proteomics. Wang *et al*. [8] elucidated the effect of Tip60 in regulating various cellular processes through the K_hib_ pathway. Although these studies have improved our understanding of K_hib_ modification at the proteomic scale, further experimental studies are required to characterize the functional role of K_hib_ in diverse cellular pathways.

Previous studies have shown that there are some specific amino acid preferences surrounding the K_hib_ site. For example, Huang *et al*. [6] found that negatively charged amino acids (i.e. aspartic acid (D) and glutamate (E)) were enriched at −1 and + 1 positions in HeLa cells, while positively charged amino acid lysine (K) was enriched at −6, −5, +5 and + 6 positions, K and arginine (R) were depleted at −1 and + 1 positions and proline (P) in most positions shows a large reduction trend. However, Huang *et al*. [9] found that in HCT116 cells, the amino acids K and R of the flanking sequence motif of K_hib_ site were enriched at −5, −6, +5, +6 positions, and the hydrophobic amino acids alanine (A) and isoleucine (I) were enriched at −1, −2, −3, −4 and + 2, and R at the −1 position was largely depleted. Meng *et al*. [10] identified 12 motifs in rice seeding leaves and found that D, E and K at −9 position, R at +8 position, valine (V) at −1 and + 2 positions were overpresented around K_hib_ sites, while K, P, R and serine (S) at position −4 to +4, were underpresented around the K_hib_ sites. Xue *et al*. [11] also detected 10 conserved motifs near the K_hib_ sites in rice seeds. Their analysis showed that D at −2, −3 positions and E at −1, −2, −3 positions, K and R at +1 position are enriched, while K and R at −1 to −4 positions are depleted. These characteristic biases imply that K_hib_ sites might have distinct characteristics for different cell types of the same species and vary among different species.

Compared with experimental methods for detecting K_hib_ sites by LC–MS, computational methods are more efficient and straightforward. To our best knowledge, to date five tools have been developed to predict K_hib_ sites. Ju *et al*. [12] used the maximum relevance and minimum redundancy method to remove the relevant and redundant features and then used the fuzzy support vector machine (SVM) to build the predictor. Wang *et al*. [13] used four different feature encoding schemes based on sequence information, physical and chemical properties and evolutionary-derived information to represent a wide range of protein sequences, and found that using SVM to build the final model can lead to the best results. The model can be used for K_hib_ site prediction of *Saccharomyces cerevisiae*, physicotrella patens, rice seeds and HeLa cells. Ju *et al*. [14] used the composition of k-spaced amino acid pairs, binary encoding and amino acid factors as the feature representation vector of amino acids, and used an ensemble SVM to build the model to predict K_hib_ sites in human HeLa cells. Zhang *et al*. [15] proposed a deep learning algorithm based on the convolutional neural networks using one-hot encoding and developed a general model based on the comprehensive data of multiple species. More recently, Bao *et al*. proposed a new algorithm to predict the K_hib_ sites for human HeLa cells, *Physcomitrella patens*, Rice seeds and *S. cerevisiae* [16].

Although the performance of the existing methods was generally good, there remains a research gap and an urgent need to develop new methods due to the following three aspects: (1) previous studies have demonstrated that the characteristics around K_hib_ sites vary among different species, as well as cell types [4–8, 10, 11, 17, 18]. However, few of currently available tools can be used to predict cell type-specific K_hib_ sites; (2) the accumulation of most recent experimental datasets allows us to develop new methods with higher accuracy, which can be applied for the identification of novel K_hib_ sites on the proteomic scale; (3) there is currently no predictor for mouse K_hib_ sites identification. We, herein, propose a predictor named ResNetKhib, which aims to identify K_hib_ sites for cell type-specific precisely. By designing and applying a residual connection of ResNet based on the one-dimensional convolution framework and the transfer learning-based training strategy, we show that ResNetKhib can predict cell type-specific K_hib_ sites with a better performance than previously reported methods. In addition, we also implement an online web server, which is publicly available at https://resnetkhib.erc.monash.edu/. We anticipate that the proposed ResNetKhib predictor can serve as a useful bioinformatic tool for effective identification of lysine K_hib_ sites and provide putative candidates to facilitate hypothesis-driven experimental validation.

## Materials and methods

### Outline of the work

In this study, we collected experimentally verified K_hib_ sites by searching the literature and constructed the benchmark datasets according to their species and cell types. As a result, 10 datasets, including eight cell type-specific and two general datasets, were curated. A detailed summary of the benchmarking and independent datasets is provided in Table 1. To develop an effective machine learning method for K_hib_ site prediction, we employed and evaluated the performance of multi-feature encoding schemes and machine learning algorithms. Eight feature encoding schemes and the word embedding method were used to represent the sequences. The random forest (RF) and one-dimensional based-ResNet algorithms were employed to integrate these encodings. Due to the excellent performance of ResNet with word embedding, we chose it as the final model to develop the ResNetKhib predictor. In addition, we also analyzed the sequence motif conservation between the K_hib_ and non-K_hib_ sites across different species and cell types, as well as the cell type-specific motifs of a species. We compared the performance of our method and state-of-the-art predictors and implemented a web server of the ResNetKhib algorithm.

**Table 1:** A statistical summary of the benchmarking and independent datasets curated

**Species**	Dataset	Organ/cell type	Benchmark dataset		Independent dataset		Ref.
			No. of positive samples	No. of negative samples	No. of positive samples	No. of negative samples	
Homo sapiens	Human_L	Lung (A549 cells)	6042	72 279	1525	18 056	[4]
	Human_U	Uterus (HeLa cells)	4433	51 480	1147	12 832	[6]
	Human_O	oral cavity (oral squamous cell carcinoma cells)	669	23 085	173	5766	[7]
	Human_K	Kidney (HEK293 cell line cells)	2443	32 560	648	8103	[8]
	Human_G	Merged	13 587	179 404	3493	44 757	[4, 6–8]
Mus musculus	Mouse_L	Liver (C57 mice)	14 361	80 363	3624	20 057	[17]
Oryza sativa	Rice_L	Leaves (Nipponbare)	2792	22 440	690	5618	[11]
	Rice_S	Seeds (Nipponbare)	6036	32 538	1522	8122	[10]
	Rice_F	Flowers (wild-type ZH11)	1829	14 264	471	3553	[5]
	Rice_G	Merged	10 657	69 242	2683	17 293	[5, 10, 11]

### Dataset collection

The experimentally verified K_hib_ sites from three species, including *Homo sapiens* (human), *Mus musculus* (mouse) and *Oryza sativa* (rice), were collected to construct the benchmark datasets. Given the amino acid usage biases in different cell types around K_hib_ sites, we constructed the datasets according to their cell types. In total, eight cell type-specific datasets and two general datasets were extracted. The four cell type-specific datasets of *H. sapiens* were collected from lung cell (A549 cells) [4], uterus cell (HeLa cells) [6], oral cavity cell (oral squamous cell carcinoma cells) [7] and kidney cell (HEK293 cells) [8], respectively; while the three cell type-specific datasets of rice were from leaves (*Nipponbare*) [11], seeds (*Nipponbare*) [10] and flowers (ZH11) [5]. For *M. musculus* data, there was only one cell type-specific dataset, which was from the liver cell (C57 mice) [17]. In addition, we also merged the cell type-specific datasets of human and rice into two general datasets. Table 1 provides a statistical summary of these datasets curated after the sequence redundancy reduction.

For each dataset, the data were preprocessed in the same manner. Here, we take the human_L dataset (i.e. human lung cell in Table 1) as an example to illustrate the preprocess procedures. A total of 2482 protein sequences of human lung cells were collected from a previous study [4] and were downloaded from Uniprot database [19] using their corresponding UniProt IDs. The CD-HIT [20, 21] program was used to remove the redundant sequences with the sequence identity of 40% [15]. In particular, for each sequence cluster in the CD-HIT result, the protein with the largest number of K_hib_ sites was selected as the representative, in which lysine sites were experimentally verified to be 2-hydroxyisobutyrylated were considered as positive samples. In contrast, the remaining lysine sites were taken as negative samples. Then, we extracted a 37-residue peptide sequence (−18 to +18) for each site, with the lysine site located at the center according to the previous study [15]. It should be noted that if the central lysine site is located near the protein sequence’s N-terminus or C-terminus, then the gap symbol ‘X’ would be assigned to fill in the corresponding positions to ensure that the peptide had the same window size. Finally, 7567 positive samples and 90 335 negative samples were obtained. Then, the whole dataset was divided into two subsets: one for 10-fold cross-validation and the other for the independent test. About 80% of the samples (i.e. 6042 positive samples and 72 279 negative samples) were subjected to 10-fold cross-validation. The remaining samples (i.e. 1525 positive samples and 18 056 negative samples) were employed as the independent test dataset. A statistical summary of the benchmarking and independent test datasets for each dataset is provided in Table 1.

### Feature encoding schemes employed

To develop an effective machine learning method for K_hib_ site prediction, multi-feature encoding schemes need to be evaluated. Accordingly, we employed eight different feature encoding schemes to predict Khib sites in previous studies [12–15], as well as the word embedding method to encode 21 types of amino acids, including the gap (‘X’). These feature encoding schemes can be grouped into four major types. The first type represents the amino acid compositions, such as the composition of *k*-spaced amino acid pairs (CKSAAP), enhanced amino acid composition (EAAC) and enhanced grouped amino acid composition (EGAAC). The second type is extracted from physicochemical properties such as amino acid index (AAindex) [22, 23], amino acid factor (AAF) and Z-scales. The third type is one-hot encoding, while the fourth is BLOSUM62 encoding, derived from the protein position-specific scoring matrices [24].

#### CKSAAP encoding

The CKSAAP feature encoding calculates the frequency of amino acid pairs separated by any *k* residues [25–28]. Taking *k* = 0 as an example, there are 400 0-spaced residue pairs (i.e. AA, AC, AD, …, YY). For instance, if the residue pair AA appears *m* times in the peptide of length *L*, the composition of the residue pair AA is equal to *m* divided by the total number of *0*-spaced residue pairs in the peptide, i.e*. m* / (*L* – *k* + 1). In the present study, the maximum *k* value was set as three, which resulted in a 1600-dimensional (400 × 4) feature vector.

#### EAAC encoding

EAAC encoding [29–32] is an improved version based on the AAC (amino acid composition) encoding, which has been widely used in the prediction of multiple PTM modification sites [29, 33, 34]. It calculates AAC in fixed-length windows, continuously sliding from each peptide’s N- to C-terminal. The following equation is used to calculate the feature vector:


(1) \begin{equation*} {\displaystyle \begin{array}{c}f\left(t,\mathrm{win}\right)=\frac{N\left(t,\mathrm{win}\right)}{N\left(\mathrm{win}\right)},t\in \left\{A,C,D,\dots, Y\right\},\\[10pt] {}\mathrm{win}\in \left\{\mathrm{window}1,\mathrm{win}\mathrm{dow}2,\dots, \mathrm{win}\mathrm{dow}33\right\}\end{array}} \end{equation*}

where *N*(*t,* win) is the number of amino acid type *t* in the sliding window win*,* and *N*(win) is the length of the peptide sequence of the sliding window *win*. Accordingly, for a sliding window size of 5, a peptide with 37 residues corresponded to 33 (=37–5 + 1) sliding windows, and its feature vector dimension was 33 × 20 (amino acids) = 480.

#### EGAAC encoding

The EGAAC encoding is based on the EAAC encoding in which the 20 amino acid types are categorized into five major groups according to their physicochemical properties, including the aliphatic group (‘GAVLMI’), aromatic group (‘FYW’), positive charge group (‘KRH’), negative charge group (‘DE’) and uncharged group (‘STCPNQ’). The frequency of each group is calculated for each sliding window. Therefore, for a sliding window size of 5, a sample with 37 residues corresponded to 33 (37–5 + 1) sliding windows and its feature vector dimension was 33 × 5 (amino acid groups) = 165.

#### A‌Aindex encoding

AAindex is a database of numerical indices representing various physicochemical and biochemical properties of amino acids [22, 23]. There are 566 physical and chemical properties collected from the AAindex database. After removing the physical and chemical properties with missing values, 553 physical and chemical properties were retained. We calculated the performance for each property using the RF classifier and selected the top 50 properties. Therefore, a peptide with 37 residues was converted to a feature vector of 37 × 50 (properties) = 1850.

#### A‌AF encoding

Using multivariate statistical analysis, amino acids’ physicochemical and biochemical properties in the AAIndex database can be transformed into five multidimensional attributes, which reflect the polarity, secondary structure, molecular volume, codon diversity and electrostatic charge, respectively [35]. Therefore, the 37 residues peptide can be encoded to a 185-dimensional (37 × 5) vector.

#### BLOSUM62 encoding

The BLOSUM62 matrix [24] is employed to represent the protein primary sequence information. A matrix comprising *m* × *L* elements represents a peptide, where *L* denotes the peptide length and *m*  =  21, which elements contain 21 amino acids, including the gap (X). Each row in the BLOSUM62 matrix is adopted to encode one of the 21 amino acids. Therefore, the dimension of the BLOSUM62 encoding is 37 × 21 = 777.

#### Z-scales encoding

For this descriptor, each amino acid is characterized by five physicochemical descriptor variables, which were proposed by Sandberg *et al*. in 1998 [36]. Therefore, a peptide with 37 residues can be encoded to a 185-dimensional (37 × 5) vector.

#### One-hot encoding

The one-hot [37, 38] encoding scheme is the most popular and easiest encoding method to transform protein sequences into numeric vectors. In the one-hot encoding, each amino acid is represented by a 21-dimensional binary vector, e.g. A is encoded by (100000000000000000000), C is encoded by (010000000000000000000), …, Y is encoded by (000000000000000000010) and X is encoded by (000000000000000000001), respectively.

### RF classifier with different features

RF [39] is a well-established and widely adopted algorithm that has been widely used in various bioinformatics studies [15, 33, 40–43]. RF is essentially an ensemble of a number of decision trees built on *N* random subsets of the training data, and the average prediction performance is often reported. In this study, we implemented the RF classifier based on the python ‘sklearn’ package [44], and the number of decision trees was set to 300.

### The ResNetKhib methodology

**Figure 1:**
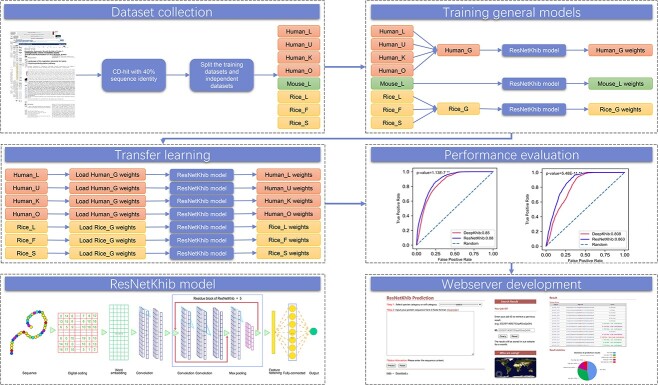
Overview of the ResNetKhib methodology.

In this work, we introduce a novel computational approach, ResNetKhib, for predicting K_hib_ sites from sequence information. ResNetKhib leverages the residual connection of ResNet based on the one-dimensional convolution framework and uses word embedding and one-hot encoding as the input to the ResNet-based models. An overview of the architecture of the proposed methodology of ResNetKhib is illustrated in Figure 1.

#### The ResNetKhib model with word embedding

**Figure 2:**
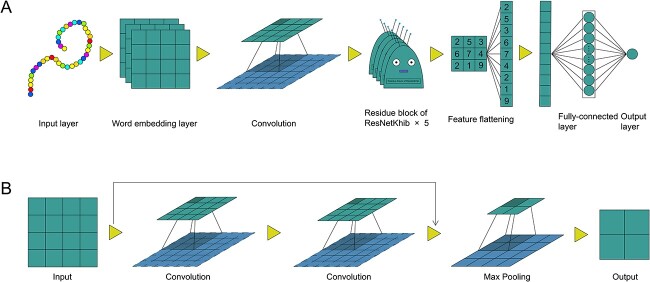
The architecture of the proposed ResNetKhib framework (**A**) and the residue block of ResNetKhib (**B**).

The architecture of ResNetKhib with word embedding (ResNetKhib_WE_) comprises the following five modules (Figure 2A):

(i) Input layer: in this layer, each peptide with 37 residues is transformed into a list of indices of length 37;
(ii) Embedding layer: each index is converted into a 512-dimensional word vector to represent the amino acid properties;
(iii) Convolution module: the convolution module contains six convolution blocks. The first block is a convolution layer with 64 filters of kernel size 1, while the remaining five are sequentially connected basic residual blocks (Figure 2B) [45];
(iv) Fully connected layer: This layer takes the output from the above layers, flattens them and converts them into a one-dimensional vector, which comprises 16 neurons;
(v) Output layer: the layer contains only one neuron, which outputs the final probability score indicating the likelihood of the lysine residue in the center to be 2-hydroxyisobutyrylated. The ‘sigmoid’ function is utilized as the activation function.

#### The ResNetKhib model with one-hot encoding

The ResNetKhib model with one-hot encoding (ResNetKhib_one-hot_) contains four modules (Supplementary Figure S1). The one-hot encoding of the 37-residue peptide was utilized as the inputs to the input layer, and there was no embedding layer in this model. The convolution module contains six convolution blocks. The first block is a convolution layer with 64 filters of kernel size 5, and the rest modules are the same as the corresponding modules in ResNetKhib_WE_.

### Model training strategy

In the present study, the parameters of ResNetKhib for general datasets (i.e. human_G and rice_G datasets in Table 1) and Mouse_L datasets were trained and optimized based on the binary cross-entropy (BCE) loss function using the Adam algorithm [46]. The maximum training cycles were set as 1000 epochs to ensure that the loss function value converged. The early stopping strategy was also used during the training process (i.e. stop training when the loss value did not decrease for 100 epochs). The training dataset was separated in each epoch with a batch size of 1024. To avoid overfitting, we added the dropout [47] operation after each convolution layer of ResNetKhib_WE_ and ResNetKhib_one-hot_, with the dropout rate set as 0.5.

The BCE loss is defined as follows:


(2) \begin{equation*} \mathrm{Loss}=-\frac{1}{N}{\sum}_{i=1}^N\left[{y}_i\log \left({p}_i\right)+\left(1-{y}_i\right)\log \left(1-{p}_i\right)\right] \end{equation*}

where *N* is the number of samples in a batch, *y* denotes the ground truth label of the samples and *p* denotes the model’s predicted value, respectively.

### Transfer learning for cell type-specific models

Transfer learning is a machine learning technique, which refers to the reuse of a pre-trained model on a new problem and can be applied to address the issue of data scarcity by leveraging existing knowledge from the source task to the target task with limited data [48]. It can help to solve the machine-learning task with limited data and improve the model performance. In this study, we applied the transfer learning strategy to train cell type-specific models in humans and rice. Taking the training process of the Human_O model as an example, the well-trained Human_G model was used as the pre-trained model. We first loaded the parameters of the pre-trained model to the ResNetKhib network and then implemented the fine-tuning technique with no parameters constrained. The transfer learning process was also trained and optimized based on the BCE loss function using the Adam algorithm, with the learning rate set to 0.01. The maximum training cycles were set as 500 epochs, and the early stopping strategy was employed to avoid overfitting. The loss-accuracy curves on each dataset are shown in the Supplementary Figure S2.

### Performance evaluation strategies

Two validation methods, including a 10-fold cross-validation test and an independent test, were used to assess the performance of the developed models. Seven performance metrics, including accuracy (Acc), sensitivity (Sn), precision (Pr), Recall (Re), specificity (Sp), F1-score and Matthew’s correlation coefficient (MCC), are adopted to evaluate the prediction performance. They are defined as follows:


(3) \begin{equation*} \mathrm{Acc}=\frac{\mathrm{TP}+\mathrm{TN}}{\mathrm{TP}+\mathrm{FN}+\mathrm{TN}+\mathrm{FP}} \end{equation*}

(4) \begin{equation*} \mathrm{Sn}=\operatorname{Re}=\frac{\mathrm{TP}}{\mathrm{TP}+\mathrm{FN}} \end{equation*}

(5) \begin{equation*} \mathrm{Sp}=\frac{\mathrm{TN}}{\mathrm{TN}+\mathrm{FP}} \end{equation*}

(6) \begin{equation*} \Pr =\frac{\mathrm{TP}}{\mathrm{TP}+\mathrm{FP}} \end{equation*}

(7) \begin{equation*} F1-\mathrm{score}=\frac{2\times \mathrm{TP}}{2\times T\mathrm{P}+\mathrm{FP}+\mathrm{FN}} \end{equation*}

(8) \begin{equation*} \mathrm{Mcc}=\frac{\mathrm{TP}\times \mathrm{TN}-\mathrm{FP}\times \mathrm{FN}}{\sqrt{\left(\mathrm{TP}+\mathrm{FP}\right)\times \left(\mathrm{TP}+\mathrm{FN}\right)\times \left(\mathrm{TN}+\mathrm{FN}\right)\times \left(\mathrm{TN}+\mathrm{FP}\right)}} \end{equation*}

where TP, FP, TN and FN represent the numbers of true positives, false positives, true negatives and false negatives, respectively. F1-score is used to assess the performance of the machine learning model, while MCC measures the differences between the prediction outputs and actual results [49]. Additionally, we also plotted the receiver operating characteristic (ROC) and precision-recall curves, calculated the area under the ROC curves (AUROC) and the area under the precision–recall curves to evaluate the performance of the predictors.

### Statistical methods

The bootstrap test [50] is typically used to assess the significance of differences between data quantified with the ROC curves. The statistical methods in this study were implemented by the iLearnPlus software toolkit [32].

## Results and discussion

### Motif conservation analysis of K_hib_ sites in different species and cell types

**Figure 3:**
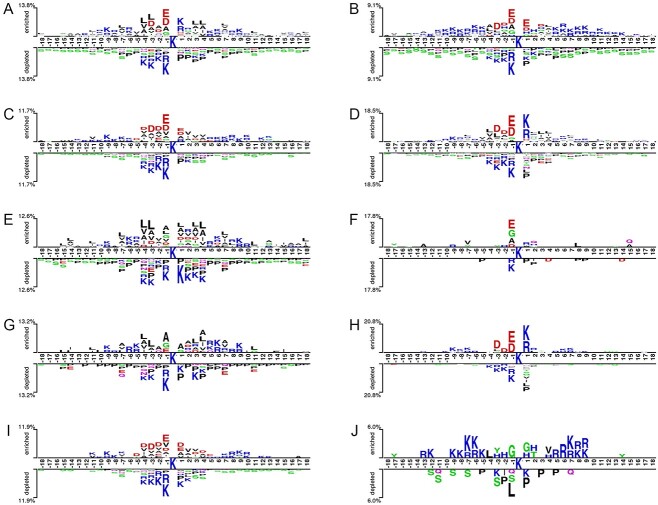
Motif conservation analysis for human_G dataset (**A**), mouse_L dataset (**B**), rice_G dataset (**C**), human_L dataset (**D**), human_U dataset (**E**), human_O dataset (**F**), human_K dataset (**G**), rice_L dataset (**H**), rice_S dataset (**I**) and rice_F dataset (**J**). The plots were generated with the Two-Sample-Logos algorithm with the *t*-test (*P-*value < 0.005).

To illustrate the distribution and preference of the flanking residues of K_hib_ sites in different species and cell types, we examined their motif conservation using the Two-Sample-Logos algorithm [51]. The logos of motif conservation for the ten datasets are presented in Figure 3. Generally, there are both parallels and differences in the sequence context around the K_hib_ sites among different species (Figure 3A–C). For example, the negatively charged amino acids D and E were significantly enriched at the −1 position, while the positively charged amino acids K and R were depleted at the −4 to −1 positions for K_hib_ sites across all three species (i.e. human, mouse and rice). However, the amino acids D and E were enriched in the +1 position for mouse and rice K_hib_ sites, while the overpresented residues at the same position for human K_hib_ sites were K and R. The amino acids V and A were enriched at the −4 to −1, +2 to +4 positions for rice K_hib_ sites. Interestingly, this pattern was not observed at the same positions for mouse K_hib_ sites.

In addition, the sequence patterns around K_hib_ sites were predominantly different among different cell types of the same species. For human, amino acids D and E were enriched in the −1 position in lung A549 cells (Figure 3D) and squamous cell carcinoma cells (Figure 3F). Amino acids A and G were also overpresented at −1 position in squamous cell carcinoma cells, while D was enriched at −3 and − 2 positions in lung A549 cells. The leucine (L), V and A were significantly enriched at −7, −4, −3, −1, +1, +3, +4 and + 7 positions for HeLa cells. However, this pattern is weakly observed in kidney HEK293 cells and lung A549 cells and is completely absent in the squamous cell carcinoma cells. The sequence patterns surrounding the K_hib_ sites across different cell types for rice appeared to be significantly different. For instance, the positively charged amino acids K and R are overpresented at +1 position in leave cells (Figure 3H) and underpresented at −4 to −1 positions in leave and seed cells. However, amino acids K and R are overpresented at −13, −12, −9 to −5, +5 to +9 positions in flower cells, and amino acids D was over-presented at −3 and − 2 positions in leave cells, as well as at −5 to −2, +1 + 3 and + 4 positions in seed cells, respectively. Such diverse patterns in K_hib_ sites among different cell types of the same species highlight the need and motivate us to develop cell type-specific predictors for K_hib_ site prediction.

To explore the cell type-specific motifs, we further compared the distribution and preference of the flanking residues between the positive samples of any two different cell types in a species (Supplementary Figure S3). In particular, for the Human_L dataset, the amino acids K and R are predominantly significantly enriched at position +1 compared with the Human_K, Human_O and Human_U datasets (Supplementary Figure S3A–C). For the Rice_L dataset, K and R are enriched at position +1 position compared with the Rice_F and Rice_S datasets (Supplementary Figure S3G and I), while K and R were are overpresented at −1 position for the Rice_F dataset compared with Rice_L and Rice_S datasets (Supplementary Figure S3G and H). The differences can be observed between any of the two positive datasets. Nevertheless, when compared with other cell types, no unified motifs can be observed for the remaining datasets (except for the Human_L, Rice_F and Rice_L datasets).

### Performance evaluation on 10-fold cross-validation and independent tests

A variety of computational approaches have been developed for the prediction of PTM sites. They are generally based on different machine learning algorithms combined with various pre-defined encoding schemes from sequences. In this section, we first evaluated the prediction performance of eight different encoding schemes using the RF classifier by conducting the 10-fold cross-validation and independent tests on the 10 datasets (Materials and Methods section). Then we compared the performance of RF-based models with ResNet-based models. Supplementary Tables S1–S10 show the performance comparison of different encoding schemes in terms of the accuracy, sensitivity, specificity, MCC and AUROC value on the 10 datasets based on the 10-fold cross-validation and independent tests. We can see that among the RF-based models, the AAindex, EAAC and EGAAC encodings performed remarkably better than the other encoding schemes on the 10 datasets. Taking the human_U dataset as an example (Supplementary Table S2), the EGAAC encoding achieved the best performance with an AUROC value of 0.789 (Acc = 87.72%; Sn = 37.63%; Sp =90.45%; MCC = 0.20) on the 10-fold cross-validation. This was followed by the EAAC encoding (AUROC = 0.783; Acc = 87.52; Sn = 39.21%; Sp = 90.15%; MCC = 0.21) and AAindex encoding (AUROC = 0.766; Acc = 87.48%; Sn = 39.92%; Sp = 90.06%; MCC = 0.21). The results on the independent test were consistent with the result based on the 10-fold cross-validation test. Then, we utilized the same strategy to assess the performance of the ResNet-based models and the top three RF-based models with the best performance (Table 2). We can see that the ResNet-based models performed obviously better than the RF-based models, and ResNetKhib_WE_ models achieved the best performance. For ResNetKhib_WE_ model, the AUROC values ranged from 0.807 to 0.901 on the 10 datasets. However, the AUROC values for RF-based models ranged from 0.525 to 0.847, indicating that the ResNet-based models could effectively capture the key characteristics underlying the K_hib_ sites for each cell type.

**Table 2:** Performance comparison of RF-based models and ResNet-based models on the 10 datasets based on 10-fold cross-validation and independent test

Datasets	RF_EAAC_	RF_EGAAC_	RF_AAindex_	ResNetKhib_WE_	ResNetKhib_Onehot_
Human_L	0.831/0.836	0.814/0.810	0.839/0.847	0.899/0.901	0.889/0.889
Human_U	0.783/0.772	0.789/0.783	0.766/0.762	0.882/0.890	0.859/0.863
Human_O	0.608/0.621	0.643/0.652	0.608/0.644	0.852/0.865	0.717/0.702
Human_K	0.757/0.775	0.776/0.764	0.738/0.745	0.881/0.851	0.848/0.819
Human_G	0.787/0.787	0.773/0.774	0.787/0.797	0.863/0.868	0.856/0.862
Mouse_L	0.786/0.785	0.758/0.754	0.77/0.769	0.852/0.851	0.848/0.848
Rice_L	0.808/0.804	0.795/0.785	0.790/0.788	0.871/0.859	0.845/0.837
Rice_S	0.740/0.753	0.734/0.746	0.724/0.724	0.827/0.832	0.803/0.813
Rice_F	0.731/0.706	0.707/0.684	0.678/0.641	0.829/0.807	0.766/0.739
Rice_G	0.714/0.716	0.702/0.708	0.710/0.706	0.811/0.809	0.791/0.791

*Note*: The result for 10-fold cross-validation and independent dataset are separated by the symbol ‘/’.

To visualize the features learned by the ResNetKhib_WE_ models, we visualized the sample distributions based on the independent dataset, from the outputs of the embedding layer, the last convolutional layer and the fully connected layer using t-SNE [52]. The results are shown in the Supplementary Figure S4. We can see that in the embedding layer, the positive and negative samples were mixed together. However, these two types of samples were clearly separated from each other after the convolutional layer and further separated after the fully connected layer. These indicate that the deep learning framework of ResNetKhib_WE_ could effectively learn the feature representations and thereby distinguish the positive from negatives samples.

### Evaluation of the prediction models on different datasets

**Figure 4:**
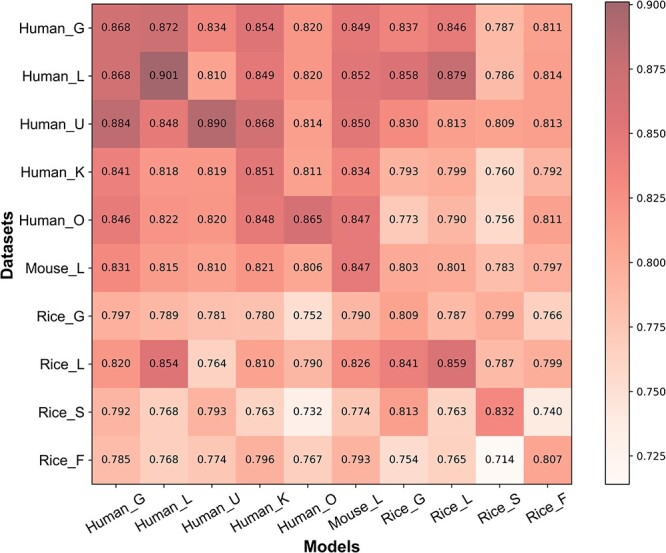
The confusion matrix of model performance on different datasets. The *x*-axis represents the datasets, while the *y*-axis denotes the models. The value in the matrix denotes the AUROC value of a model on the corresponding dataset.

In this study, we trained the model using each dataset based on the ResNetKhib_WE_ framework. The confusion matrix of AUROC values for models on different datasets is shown in Figure 4, where the *x*-axis denotes the models, while the *y*-axis represents the datasets. The value in the confusion matrix denotes the performance of a model on the corresponding dataset. As can be seen, for each cell type-specific dataset, the diagonal position has the highest AUROC value, indicating that the cell type-specific models performed better than the general models. For instance, the human general model achieved an AUROC value of 0.868 on the human_L dataset, while the cell type-specific model further improved the AUROC value to 0.901. We also compared the performance of cell type-specific models trained without the transfer learning strategy (Table 3). For the majority of cell type-specific datasets, the transfer learning strategy can improve their prediction performance, especially for the datasets with a small sample size. Taking the human_O dataset as an example, there were only 669 samples human_O dataset, which is obviously insufficient to train a model. The AUROC value was improved from 0.826 to 0.865 using the transfer learning strategy on the human_O independent test dataset, suggesting that transfer learning could not only accelerate the training process but also improve the prediction performance of the model. Moreover, to further evaluate the robustness of ResNetKhib, we also rebuilt the training dataset with the 30% sequence identity and retrained the models. As can be seen from the Supplementary Table S11, the performance of seven retrained models decreased slightly, while the performance of three retrained models improved compared with the models trained on the datasets with the 40% sequence identity.

**Table 3:** Performance comparison of the model training strategy based on 10-fold cross-validation and independent test

Datasets	10-fold cross-validation		Independent test	
	Training without transfer learning strategy	Training with transfer learning strategy	Training without transfer learning strategy	Training with transfer learning strategy
Human_L	0.899	0.899	0.899	0.901
Human_U	0.878	0.882	0.884	0.890
Human_O	0.822	0.852	0.826	0.865
Human_K	0.864	0.881	0.846	0.851
Rice_L	0.865	0.871	0.852	0.859
Rice_S	0.827	0.827	0.832	0.832
Rice_F	0.822	0.829	0.796	0.807

### Performance comparison between ResNetKhib and the state-of-art predictors

**Figure 5:**
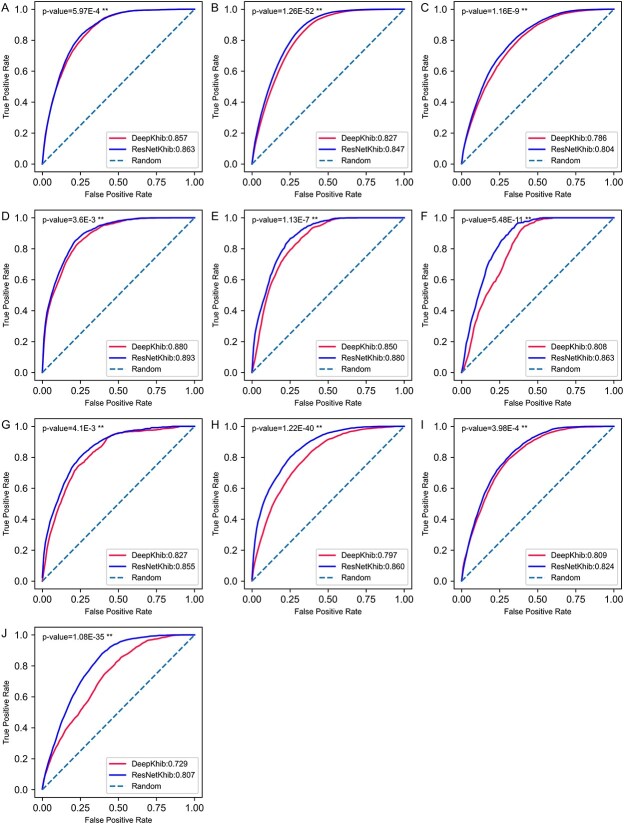
Performance comparison of the proposed ResNetKhib and the state-of-the-art predictor DeepKhib for the human_G dataset (**A**), mouse_L dataset (**B**), rice_G dataset (**C**), human_L dataset (**D**), human_U dataset (**E**), human_O dataset (**F**), human_K dataset (**G**), rice_L dataset (**H**), rice_S dataset (**I**) and rice_F dataset (**J**).

We chose the ResNetKhib_WE_ models as the final prediction models. To illustrate the predictive capability and robustness of ResNetKhib, we further compared the performance of ResNetKhib with other existing K_hib_ site predictors on the independent test datasets (for details, refer to the Material and Methods section). To date, four predictors have been developed to predict K_hib_ sites [12–15]. However, only DeepKhib [15], which includes both species-specific and general models, is currently available. To make a fair comparison, we removed the sequences from the independent test datasets that were used in DeepKhib’s training dataset. As a result, ResNetKhib outperformed DeepKhib on all datasets (Table 4, Figure 5 and Supplementary Figure S5), highlighting the importance and necessity of developing cell type-specific predictors for K_hib_ site prediction. For example, for the human_K dataset (i.e. Kidney HEK293 cell line cells), DeepKhib achieved an AUROC value of 0.827, while ResNetKhib achieved an AUROC value of 0.855 (Figure 5G). In terms of the performance with a low false positive rate (i.e. Sp = 90%), ResNetKhib identified 51.82% K_hib_ sites, whereas DeepKhib accurately predicted only 43.70% of K_hib_ sites. In addition, the *P-*values were also calculated to examine if the performance comparison between ResNetKhib and DeepKhib was statistically significant or not. As can be seen from Figure 5, ResNetKhib significantly outperformed DeepKhib across all the 10 datasets (i.e*. P-*value < 0.01), which suggests the predictive capability and robustness of ResNetKhib.

**Table 4:** Prediction performance of ResNetKhib and DeepKhib in terms of five major performance metrics, that is, Acc, Sn, Sp, F1-score, MCC

Datasets	DeepKhib					ResNetKhib				
	Acc (%)	Sn (%)	Sp (%)	MCC	F1-score	Acc (%)	Sn (%)	Sp (%)	MCC	F1-score
Human_L	89.39	57.38	90.09	0.221	0.190	89.81	60.72	90.45	0.241	0.204
Human_U	89.27	47.59	90.24	0.182	0.167	89.40	54.41	90.20	0.214	0.189
Human_O	88.33	34.19	90.18	0.141	0.162	88.62	45.88	90.07	0.204	0.209
Human_K	87.86	43.70	90.16	0.231	0.263	88.24	51.82	90.14	0.282	0.305
Human_G	89.43	49.70	90.47	0.207	0.194	88.03	50.43	89.01	0.201	0.186
Mouse_L	82.50	40.32	90.12	0.311	0.413	83.30	44.54	90.30	0.351	0.449
Rice_L	85.34	40.32	90.02	0.265	0.341	86.87	56.38	90.04	0.387	0.447
Rice_S	86.40	39.28	90.01	0.232	0.292	86.65	42.50	90.03	0.255	0.311
Rice_F	83.67	28.52	90.28	0.181	0.272	84.32	34.91	90.24	0.235	0.322
Rice_G	85.81	35.67	90.49	0.227	0.300	85.89	38.86	90.28	0.249	0.319

Taken together, we conclude that ResNetKhib achieved a remarkable performance presumably due to the following three main factors: (1) our ResNetKhib predictor was designed particularly for predicting cell type-specific K_hib_ sites; (2) the ResNet framework is more suitable for modeling the K_hib_ sites data; and (3) the training strategies of transfer learning provide the model with an excellent initial searching space, and accordingly, it could achieve a better performance compared the other predictors.

### Overlap of cell type-specific K_hib_ modified proteins and K_hib_ sites

In this section, we plotted the Venn diagrams and analyzed the overlap among different cell types of the same species at the protein and residue levels. As shown in Supplementary Figure S6, only 51 K_hib_ sites overlapped in all the four human cell types (Supplementary Figure S6A). In contrast, there were 170 K_hib_ sites appearing in all the three rice cell types (Supplementary Figure S6C). Interestingly, the number of overlapped 2-hydroxyisobutyrylated proteins was larger than that of overlapped K_hib_ sites in the four human cell types (Supplementary Figure S6A and B), indicating that the majority of the K_hib_ sites are different in number in the same protein of different human cell types. Taking the EIF3A protein (UniProt ID: Q14152) as an example, seven lysine residues of EIF3A can be 2-hydroxyisobutyrylated. The lysines K100 and K694 were 2-hydroxyisobutyrylated in the kidney cell, while K23, K742 and K775 were 2-hydroxyisobutyrylated in the lung cell. No modified K_hib_ sites were detected in squamous cell carcinoma cells, while K538 and K711 were found to be 2-hydroxyisobutyrylated in HeLa cells. For rice, the rice_S dataset has the largest number of 2-hydroxyisobutyrylated proteins, followed by rice_L and rice_F (Supplementary Figure S6D).

In order to test the predictive capability of ResNetKhib, we applied its model to predict the cell type-specific K_hib_ sites in rice. We randomly selected 100 protein sequences from the rice (*O. sativa* subsp*. japonica*) proteome, whose sequences did not overlap with those in the training dataset. We submitted these sequences to the ResNetKhib web server with the ‘HIGH’ confidence. Among these 100 protein sequences, 50, 57 and 52 protein sequences were predicted to be 2-hydroxyisobutyrylated in rice flower, leaves and seeding cells, respectively. Only seven sites and 31 proteins were predicted to be 2-hydroxyisobutyrylated in all the three cell types (Supplementary Figure S6E and F). Taking the CDKE-1 protein (UniProt ID: Q336M2) as an example, the K59 residue was predicted to be 2-hydroxyisobutyrylated in rice flower cells, while K59 and K259 were predicted to be 2-hydroxyisobutyrylated in leaves. There were no residues predicted to be 2-hydroxyisobutyrylated in the seeding cell. The results suggest that ResNetKhib can be exploited as a useful tool for predicting putative K_hib_ modified proteins and K_hib_ sites in the cell type-specific manner.

### Implementation of the ResNetKhib web server

**Figure 6:**
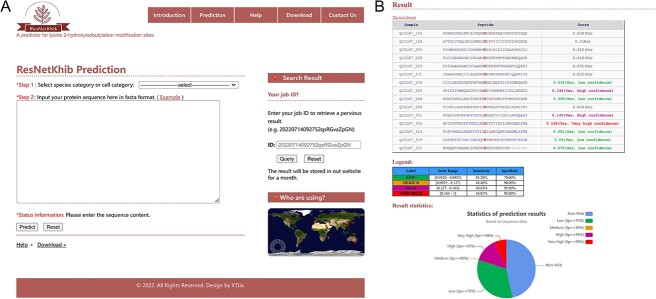
Screenshots of the developed ResNetKhib web server for prediction K_hib_ sites. (**A**) The main page of the ResNetKhib web server and (**B**) the example prediction output of the web server.

As an implementation of the proposed methodology, we have developed a user-friendly web server, which can be freely accessed at https://resnetkhib.erc.monash.edu/. Generally, for a typical protein sequence with approximately 500 amino acid residues, accomplishing a single prediction task takes about 5 s. At the prediction webpage, users can input one or more protein sequences in the textbox in the FASTA format (the maximum number of sequences allowed for a single submission is 100) (Figure 6A). To control the false-positive rate, four different cut-off values are provided (i.e. ‘VERY HIGH’ = 98% Sp, ‘HIGH’ = 95% Sp, ‘MEDIUM’ = 90% Sp and ‘LOW’ = 70% Sp). ResNetKhib web server contains 10 models, including two general models and eight cell type-specific models. Given a model of interest, users can select the suitable model to make the prediction. The predicted results can be directly displayed on the web page or downloaded in plain text format to facilitate users’ subsequent analysis (Figure 6B). At the same time, all data and corresponding models used in this study can be downloaded from the download webpage at the ResNetKhib web server.

## Conclusions

In this study, we first collected the experimentally verified K_hib_ sites from three species to construct two general and eight cell type-specific benchmark datasets. Using these datasets, we comprehensively evaluated the performance of eight feature encoding schemes based on the RF classifier and found that three encoding schemes, including the EAAC, EGAAC and AAindex encodings, performed better than the other encoding schemes. Then, we examined the distribution and preference of the flanking residues of K_hib_ sites on different species and cell types and observed that the sequence patterns around K_hib_ sites were predominantly different among different cell types of the same species. Based on our findings and inspired by the residual block of the ResNet, we constructed a deep learning network classifier called ResNetKhib based on one-dimensional convolution for the prediction of cell type-specific 2-hydroxyisobutylation sites and utilized a transfer learning strategy to train the cell type-specific models. Benchmarking tests illustrate that transfer learning could improve the model performance, especially for datasets with small sample sizes. Although four tools have been developed to predict K_hib_ sites to date, none of these tools were designed to predict K_hib_ sites in the cell type-specific manner. ResNetKhib is capable of identifying cell type-specific K_hib_ sites with a better performance. As an implementation of ResNetKhib, an online web server has been made freely accessible at https://resnetkhib.erc.monash.edu/ for the wider research community to use.

**Key Points**

- Lysine 2-hydroxyisobutylation play important roles in a myriad of diverse biological processes. Previous studies have shown that K_hib_ sites may have distinct characteristics for different celltypes of the same species. However, there are no tools designed for predicting cell type-specificKhib sites. This study aimed to predict K_hib_ sites in cell type-specific.
- We propose a new deep learning model, termed ResNetKhib, which leverages both the one- dimensional convolution and transfer learning to enable and improve the prediction of cell type-specific 2-hydroxyisobutylation sites. Experimental results demonstrate the superiorperformance of ResNetKhib compared to existing methods.
- We also benchmark its performance against the commonly used random forest predictor byperforming both 10-fold cross-validation and independent tests. The results show thatResNetKhib achieves a better performance than RF-based predictors.
- A web server (https://resnetkhib.erc.monash.edu) has been made available to facilitate onlinehigh-throughput prediction of K_hib_ sites.

## Supplementary Material

Supplementary_Figures_bbad063

Supplementary_Tables_bbad063

Response_letter_Final_bbad063

## Data Availability

The datasets used to train and evaluate ResNetKhib model are publicly accessible at ResNetKhib web server.

## Notes

**Xiaoti Jia** is a master degree candidate student at the Collaborative Innovation Center of Henan Grain Crops, and Key Laboratory of Rice Biology in Henan Province, College of Agronomy, Henan Agricultural University, China. His research interests include protein bioinformatics, machine learning and next-generation sequencing analysis.

**Pei Zhao** is an assistant professor at the State Key Laboratory of Cotton Biology, Institute of Cotton Research of Chinese Academy of Agricultural Sciences, Anyang 455 000, Henan, China and Zhengzhou Research Base, State Key Laboratory of Cotton Biology, School of Agricultural Sciences, Zhengzhou University, Zhengzhou, China. Her research interests include bioinformatics and analysis of next-generation sequencing in plants.

**Fuyi Li** received his PhD in Bioinformatics from Monash University, Australia. He is currently a professor at the College of Information Engineering, Northwest A&F University, China. His research interests include bioinformatics, computational biology, machine learning and deep learning.

**Zhaohui Qin** is a master degree candidate student at the Collaborative Innovation Center of Henan Grain Crops, and Key Laboratory of Rice Biology in Henan Province, College of Agronomy, Henan Agricultural University, China. His research interests include protein bioinformatics and deep learning.

**Haoran Ren** is a master degree candidate student at the Collaborative Innovation Center of Henan Grain Crops, and Key Laboratory of Rice Biology in Henan Province, College of Agronomy, Henan Agricultural University, China. His research interests include protein bioinformatics and deep learning.

**Junzhou Li** is a professor at the Collaborative Innovation Center of Henan Grain Crops, and Key Laboratory of Rice Biology in Henan Province, College of Agronomy, Henan Agricultural University, China. His research interests include plant molecular genetics and bioinformatics.

**Chunbo Miao** is a professor at the Collaborative Innovation Center of Henan Grain Crops, and Key Laboratory of Rice Biology in Henan Province, College of Agronomy, Henan Agricultural University, China. His research interests include plant molecular genetics.

**Quanzhi Zhao** is a professor at the Collaborative Innovation Center of Henan Grain Crops, College of Agronomy, and Key Laboratory of Rice Biology in Henan Province, Henan Agricultural University, China. His research focuses on elucidating the mechanisms of the adaptations of rice to environmental stresses and yield characters using molecular, omics and bioinformatics approaches.

**Tatsuya Akutsu** received his DEng degree in information engineering in 1989 from the University of Tokyo, Japan. Since 2001, he has been a professor in the Bioinformatics Center, Institute for Chemical Research, Kyoto University, Japan. His research interests include bioinformatics and discrete algorithms.

**Gensheng Dou** is an associate professor at the College of Agronomy, Henan Agricultural University. He is engaged in teaching and research in the direction of smart agriculture. His research interests include artificial intelligence and agricultural internet of things.

**Zhen Chen** is a professor at the Collaborative Innovation Center of Henan Grain Crops, Key Laboratory of Rice Biology in Henan Province and Center for Crop Genome Engineering, College of Agronomy, Henan Agricultural University, China. His research interests include protein bioinformatics, machine learning and analysis of next-generation sequencing data.

**Jiangning Song** is currently an associate professor and group leader in the Monash Biomedicine Discovery Institute, Monash University. He is also a member of the Monash Data Futures Institute. His research interests include bioinformatics, computational biology, machine learning, medical imaging and pattern recognition.
