# Supplementary material for: ResNetKhib: a novel cell type-specific tool for predicting lysine 2-hydroxyisobutylation sites via transfer learning
Source: Brief Bioinform. 2023 Mar 4;24(2):bbad063. doi: 10.1093/bib/bbad063 (PMC10185920; doi:10.1093/bib/bbad063)
Supplement: Supplementary_Figures_bbad063 [file supplementary_figures_bbad063.docx]

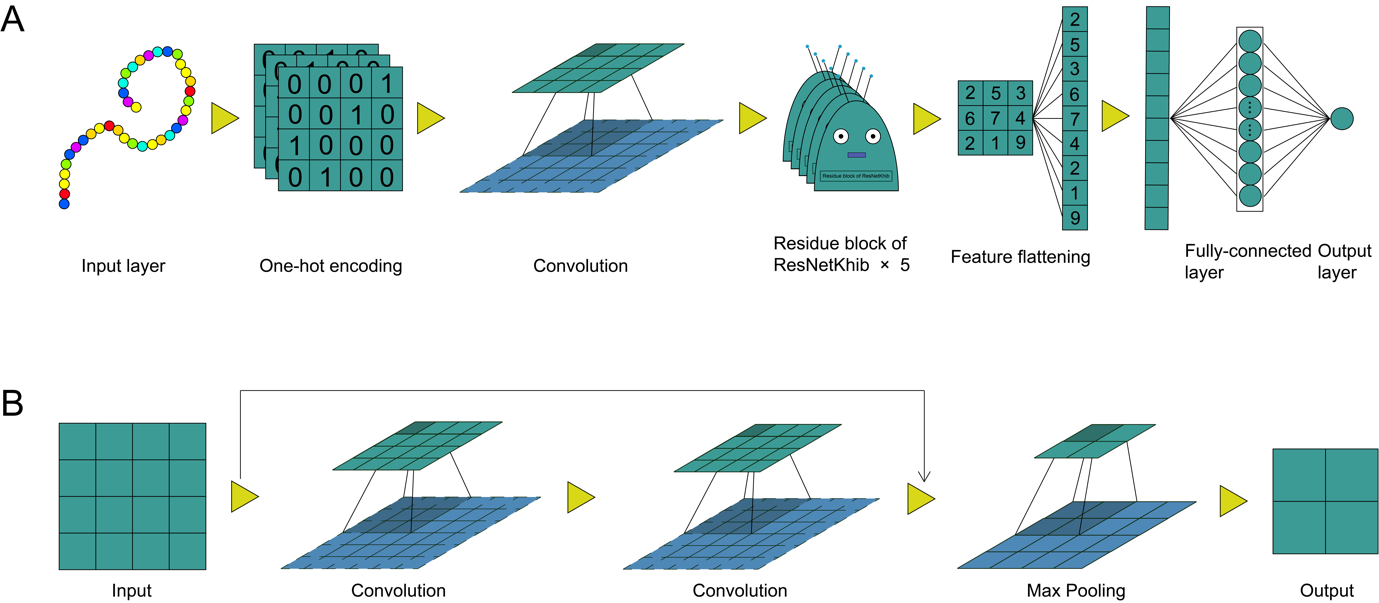


**Supplementary Figure S1. The architecture of ResNetKhib_one-hot_.**


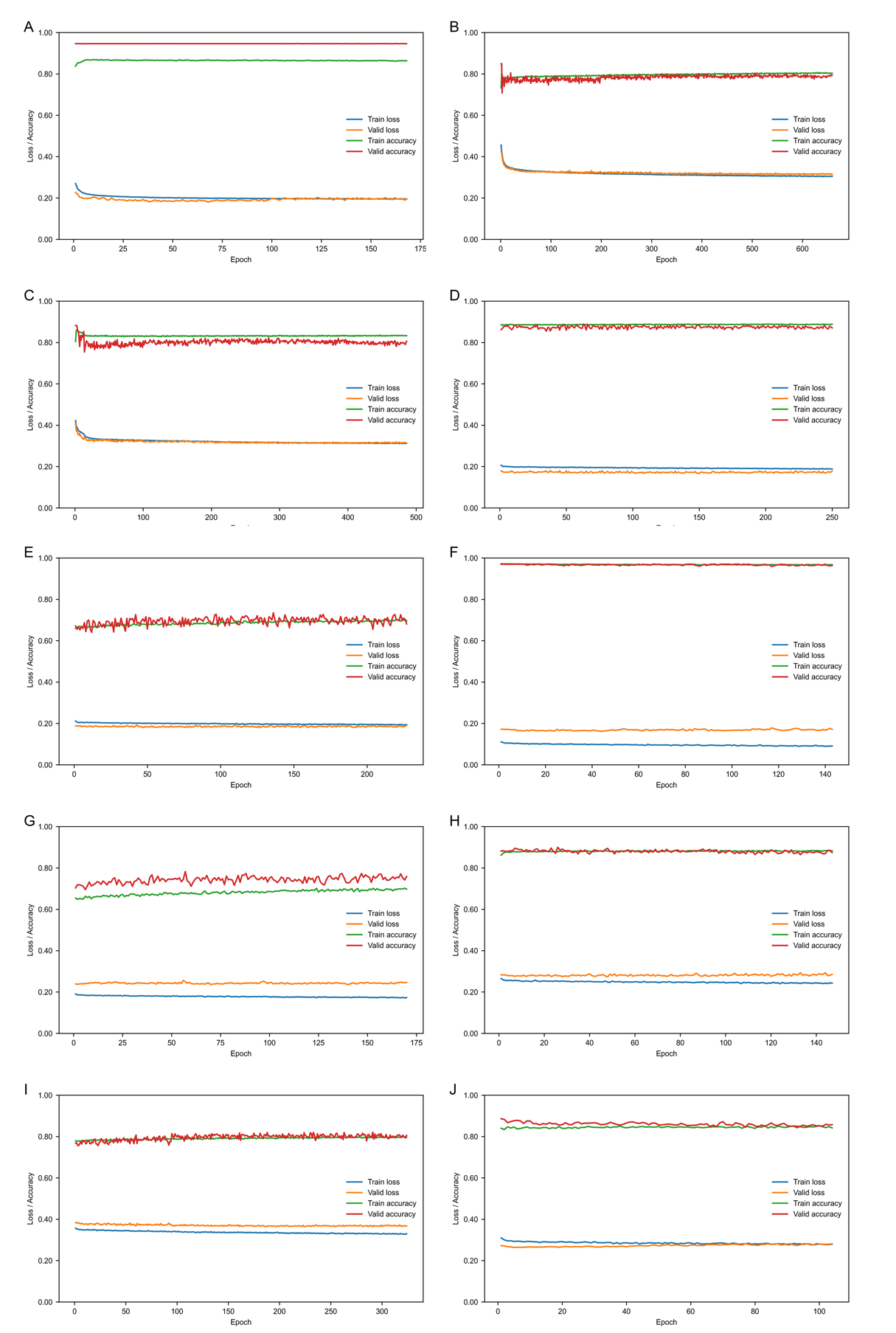


**Supplementary Figure S2. The loss-accuracy curves of the model training process for the human_G dataset (A), mouse_L dataset (B), rice_G dataset (C), human_L dataset (D), human_U dataset (E), human_O dataset (F), human_K dataset (G), rice_L dataset (H), rice_S dataset (I), and rice_F dataset (J).**


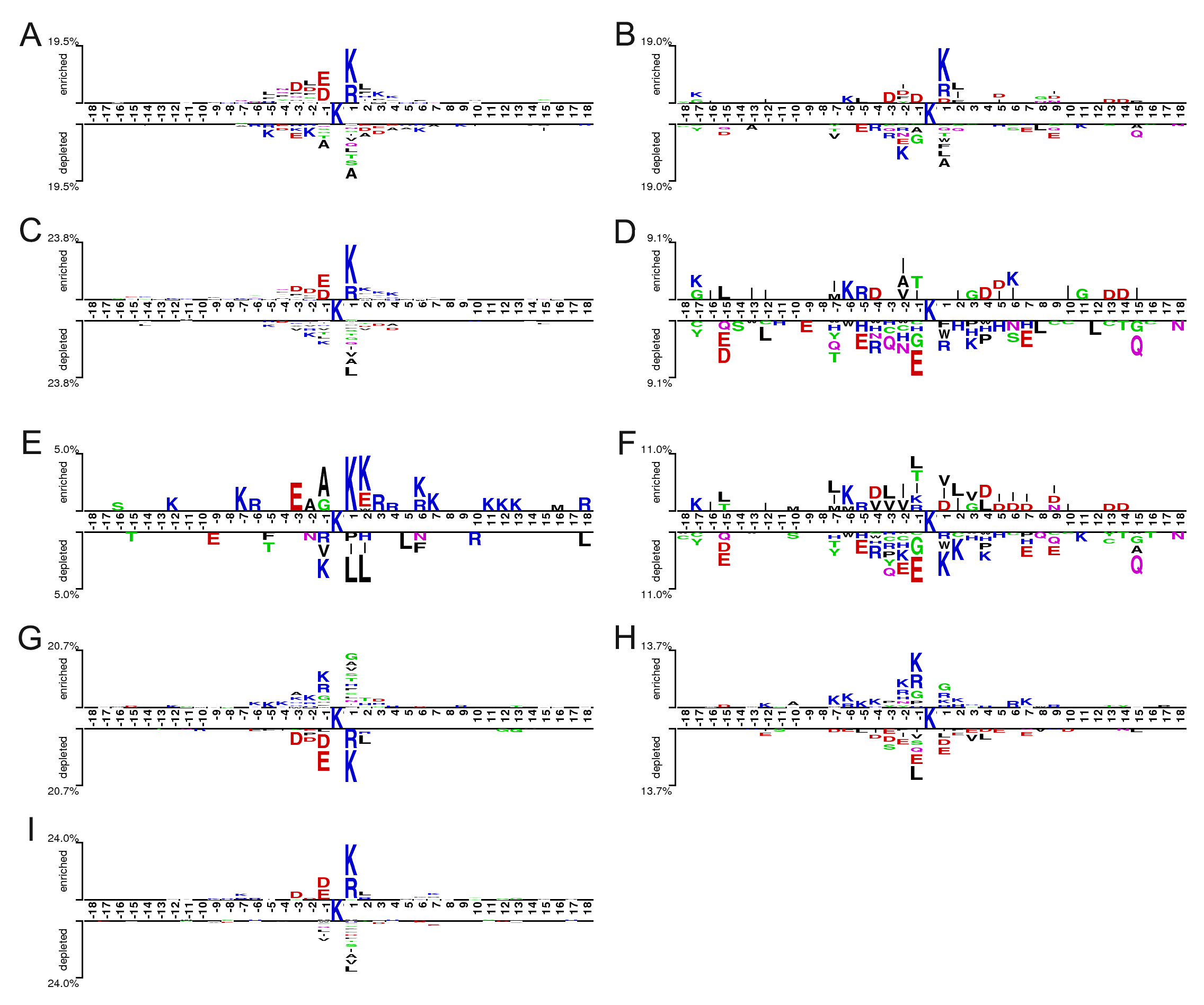


**Supplementary Figure S3. Motif conservation analysis for the positive samples between the human_L and human_K dataset (A), human_L and human_O dataset (B), human_L and human_U dataset (C), human_K and human_O dataset (D), human_K and human_U dataset (E), human_U and human_O dataset (F), rice_F and rice_L dataset (G), rice_F and rice_S dataset (H), rice_L, and rice_S dataset (I).**


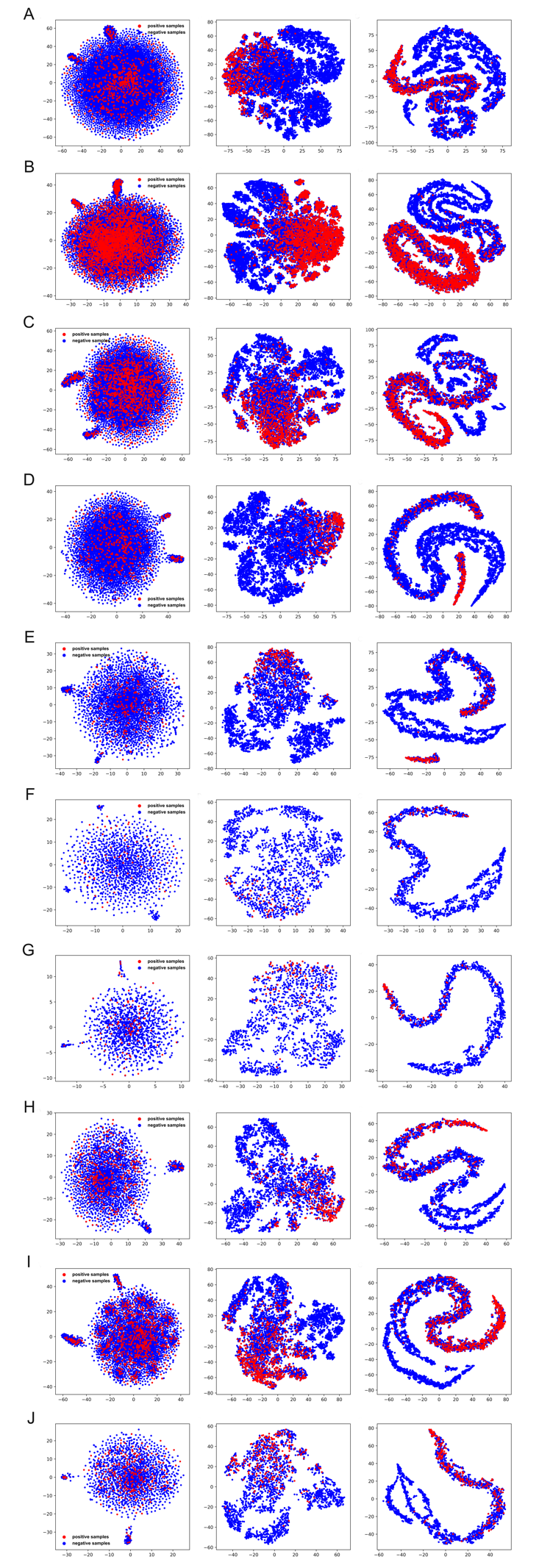


**Supplementary Figure S4. T-SNE visualization of the distributions of samples in the independent dataset for the output of the embedding layer (left), last convolutional layer (middle) and fully connected layer (right) for the human_G dataset (A), mouse_L dataset (B), rice_G dataset (C), human_L dataset (D), human_U dataset (E), human_O dataset (F), human_K dataset (G), rice_L dataset (H), rice_S dataset (I), and rice_F dataset (J).**


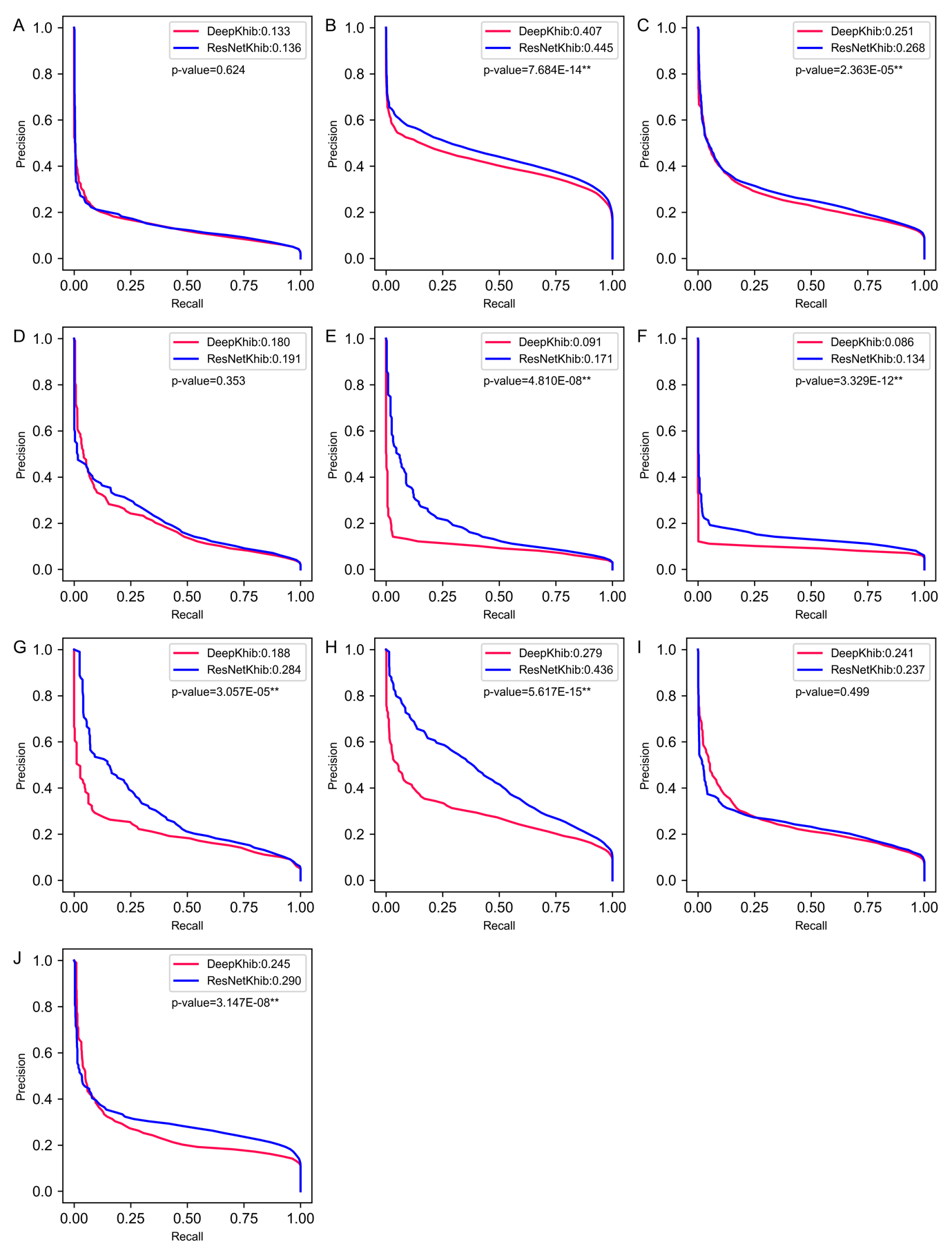


**Supplementary Figure S5. Precision-recall curves of the proposed ResNetKhib and the state-of-the-art predictor DeepKhib for the human_G dataset (A), mouse_L dataset (B), rice_G dataset (C), human_L dataset (D), human_U dataset (E), human_O dataset (F), human_K dataset (G), rice_L dataset (H), rice_S dataset (I), and rice_F dataset (J).**


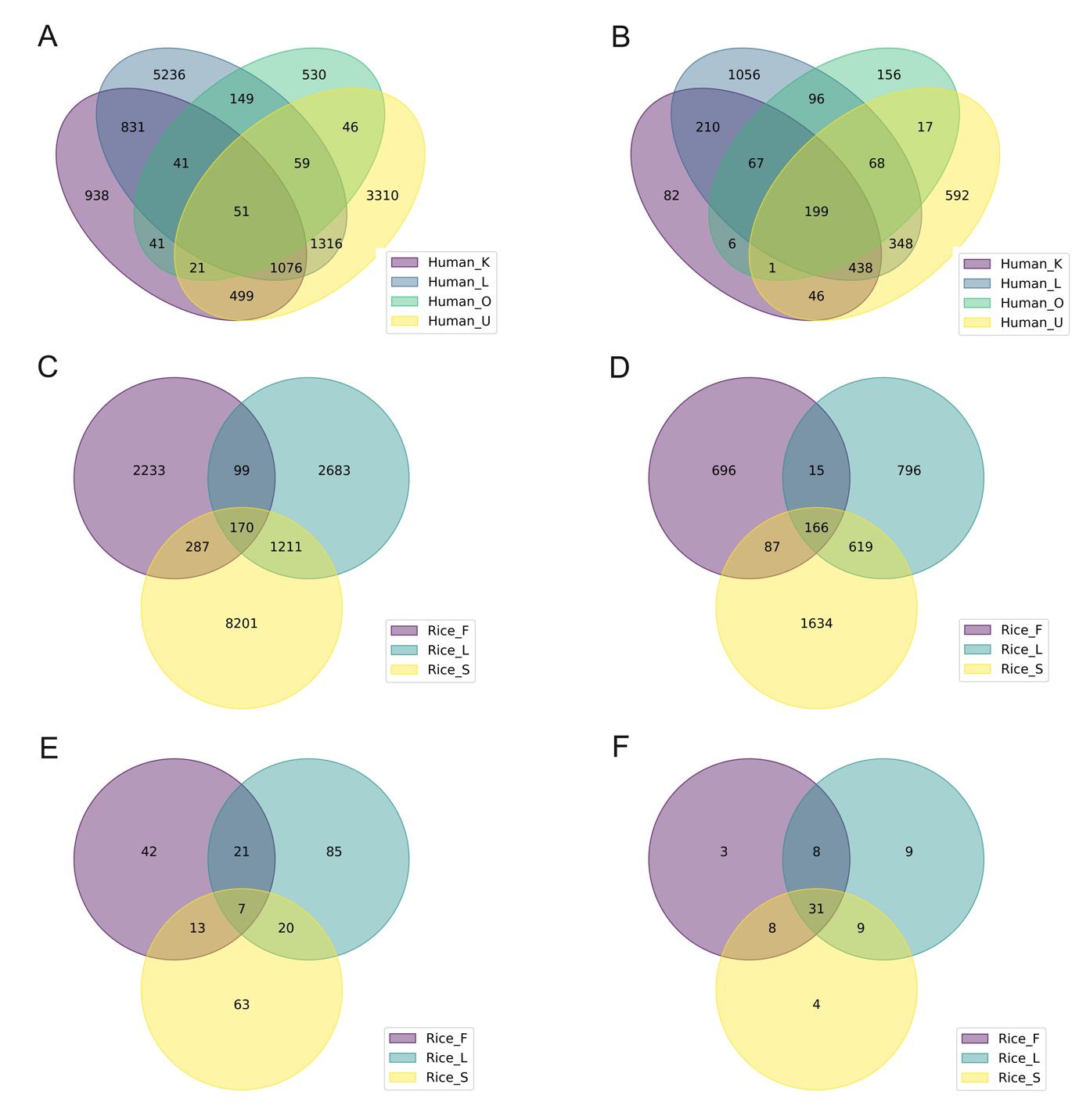


**Supplementary Figure S6. Venn diagrams showing the overlap of K_hib_ sites for the four human cell types (A), overlap of K_hib_ proteins for the four human cell types (B), overlap of K_hib_ sites for the three rice cell types (C), overlap of K_hib_ proteins for the three rice cell types (D), overlap of predicted K_hib_ sites for the three rice cell types (E) and overlap of predicted K_hib_ proteins for the three rice cell types (F).**
