# Supplementary material for: ResNetKhib: a novel cell type-specific tool for predicting lysine 2-hydroxyisobutylation sites via transfer learning
Source: Brief Bioinform. 2023 Mar 4;24(2):bbad063. doi: 10.1093/bib/bbad063 (PMC10185920; doi:10.1093/bib/bbad063)
Supplement: Supplementary_Tables_bbad063 [file supplementary_tables_bbad063.docx]

**Table S1. Performance comparison of different encoding schemes on human_L dataset based on 10-fold cross-validation and independent test.**

| **Encodings** | **Acc(%)** | **Sn(%)** | **Sp(%)** | **MCC** | **F1-Score** | **AUPRC** | **AUROC** |
| --- | --- | --- | --- | --- | --- | --- | --- |
| CKSAAP | 87.24 / 86.93 | 26.51 / 24.48 | 90.38 / 90.04 | 0.119 / 0.1 | 0.17 / 0.151 | 0.089 / 0.08 | 0.606 / 0.598 |
| One-hot | 88.22 / 88.5 | 49.73 / 51.8 | 90.21 / 90.33 | 0.268 / 0.279 | 0.293 / 0.299 | 0.218 / 0.212 | 0.798 / 0.8 |
| Z-scales | 88.34 / 88.44 | 53.42 / 55.43 | 90.15 / 90.08 | 0.29 / 0.297 | 0.311 / 0.313 | 0.248 / 0.254 | 0.812 / 0.814 |
| AAF | 88.57 / 88.84 | 52.26 / 53.39 | 90.45 / 90.6 | 0.288 / 0.293 | 0.31 / 0.312 | 0.24 / 0.235 | 0.813 / 0.811 |
| BLOSUM62 | 88.6 / 88.7 | 51.66 / 54.48 | 90.51 / 90.41 | 0.285 / 0.297 | 0.308 / 0.314 | 0.234 / 0.252 | 0.813 / 0.82 |
| EGAAC | 88.22 / 88.17 | 50.22 / 49.91 | 90.19 / 90.07 | 0.271 / 0.263 | 0.295 / 0.286 | 0.223 / 0.219 | 0.814 / 0.81 |
| EAAC | 88.69 / 88.99 | 51.9 / 53.7 | 90.59 / 90.75 | 0.288 / 0.298 | 0.311 / 0.317 | 0.254 / 0.247 | 0.831 / 0.836 |
| Aaindex | 88.53 / 88.82 | 56.27 / 57.71 | 90.2 / 90.37 | 0.309 / 0.315 | 0.326 / 0.329 | 0.274 / 0.294 | 0.839 / 0.847 |

Note: the result for 10-fold cross-validation and independent dataset are seperated by the symbol "/".

**Table S2. Performance comparison of different encoding schemes on human_U dataset based on 10-fold cross-validation and independent test.**

| **Encodings** | **Acc(%)** | **Sn(%)** | **Sp(%)** | **MCC** | **F1-Score** | **AUPRC** | **AUROC** |
| --- | --- | --- | --- | --- | --- | --- | --- |
| CKSAAP | 87.66 / 87.44 | 31.97 / 27.73 | 90.71 / 90.53 | 0.164 / 0.13 | 0.212 / 0.179 | 0.125 / 0.097 | 0.686 / 0.662 |
| One-hot | 87.88 / 88.12 | 28.7 / 30.14 | 91.1 / 91.12 | 0.147 / 0.154 | 0.197 / 0.2 | 0.135 / 0.126 | 0.716 / 0.73 |
| BLOSUM62 | 87.67 / 87.61 | 30.51 / 30.94 | 90.78 / 90.55 | 0.155 / 0.152 | 0.204 / 0.197 | 0.144 / 0.137 | 0.724 / 0.725 |
| Z-scales | 87.1 / 87.61 | 33.88 / 33.82 | 90.01 / 90.39 | 0.168 / 0.169 | 0.214 / 0.212 | 0.161 / 0.14 | 0.74 / 0.737 |
| AAF | 87.5 / 87.57 | 35.99 / 34.71 | 90.32 / 90.31 | 0.186 / 0.174 | 0.23 / 0.216 | 0.155 / 0.144 | 0.742 / 0.739 |
| Aaindex | 87.48 / 87.13 | 39.92 / 39.58 | 90.06 / 89.59 | 0.209 / 0.195 | 0.249 / 0.232 | 0.17 / 0.156 | 0.766 / 0.762 |
| EAAC | 87.52 / 87.63 | 39.21 / 34.99 | 90.15 / 90.35 | 0.206 / 0.176 | 0.246 / 0.218 | 0.167 / 0.138 | 0.783 / 0.772 |
| EGAAC | 87.72 / 87.16 | 37.63 / 37.0 | 90.45 / 89.76 | 0.199 / 0.181 | 0.24 / 0.221 | 0.172 / 0.143 | 0.789 / 0.783 |

Note: the result for 10-fold cross-validation and independent dataset are seperated by the symbol "/".

**Table S3. Performance comparison of different encoding schemes on human_O dataset based on 10-fold cross-validation and independent test.**

| **Encodings** | **Acc(%)** | **Sn(%)** | **Sp(%)** | **MCC** | **F1-Score** | **AUPRC** | **AUROC** |
| --- | --- | --- | --- | --- | --- | --- | --- |
| CKSAAP | 88.57 / 88.65 | 12.81 / 10.86 | 91.38 / 91.52 | 0.027 / 0.016 | 0.073 / 0.064 | 0.047 / 0.041 | 0.529 / 0.525 |
| AAF | 87.92 / 88.31 | 16.0 / 11.35 | 90.58 / 91.15 | 0.043 / 0.016 | 0.088 / 0.064 | 0.05 / 0.046 | 0.575 / 0.603 |
| One-hot | 87.43 / 87.44 | 16.25 / 13.76 | 90.05 / 90.15 | 0.04 / 0.024 | 0.086 / 0.072 | 0.053 / 0.044 | 0.578 / 0.581 |
| Z-scales | 87.81 / 87.63 | 18.06 / 14.61 | 90.39 / 90.31 | 0.054 / 0.031 | 0.097 / 0.077 | 0.054 / 0.048 | 0.584 / 0.605 |
| BLOSUM62 | 88.6 / 88.93 | 13.33 / 15.55 | 91.39 / 91.63 | 0.032 / 0.048 | 0.077 / 0.091 | 0.049 / 0.054 | 0.584 / 0.642 |
| EAAC | 87.98 / 87.56 | 11.51 / 18.21 | 90.81 / 90.12 | 0.016 / 0.051 | 0.065 / 0.094 | 0.049 / 0.052 | 0.608 / 0.621 |
| Aaindex | 87.87 / 87.86 | 16.83 / 20.29 | 90.49 / 90.35 | 0.046 / 0.066 | 0.09 / 0.106 | 0.052 / 0.059 | 0.608 / 0.644 |
| EGAAC | 88.28 / 88.1 | 17.92 / 12.99 | 90.9 / 90.86 | 0.055 / 0.025 | 0.097 / 0.072 | 0.067 / 0.052 | 0.643 / 0.652 |

Note: the result for 10-fold cross-validation and independent dataset are seperated by the symbol "/".

**Table S4. Performance comparison of different encoding schemes on human_K dataset based on 10-fold cross-validation and independent test.**

| **Encodings** | **Acc(%)** | **Sn(%)** | **Sp(%)** | **MCC** | **F1-Score** | **AUPRC** | **AUROC** |
| --- | --- | --- | --- | --- | --- | --- | --- |
| CKSAAP | 86.6 / 87.44 | 30.61 / 34.58 | 90.05 / 90.94 | 0.152 / 0.2 | 0.208 / 0.255 | 0.137 / 0.165 | 0.663 / 0.67 |
| Z-scales | 86.51 / 86.78 | 20.86 / 28.83 | 90.51 / 90.63 | 0.088 / 0.152 | 0.151 / 0.213 | 0.122 / 0.142 | 0.672 / 0.694 |
| One-hot | 87.33 / 87.75 | 24.51 / 26.71 | 91.16 / 91.8 | 0.125 / 0.154 | 0.183 / 0.214 | 0.132 / 0.15 | 0.68 / 0.691 |
| BLOSUM62 | 87.35 / 87.07 | 25.78 / 27.13 | 91.11 / 91.04 | 0.133 / 0.146 | 0.191 / 0.207 | 0.136 / 0.141 | 0.683 / 0.698 |
| AAF | 86.55 / 87.45 | 25.24 / 29.41 | 90.3 / 91.3 | 0.118 / 0.167 | 0.178 / 0.226 | 0.133 / 0.15 | 0.691 / 0.704 |
| Aaindex | 86.97 / 87.05 | 31.68 / 30.96 | 90.34 / 90.78 | 0.164 / 0.17 | 0.218 / 0.229 | 0.161 / 0.148 | 0.738 / 0.745 |
| EAAC | 87.55 / 88.0 | 33.48 / 33.19 | 90.86 / 91.64 | 0.187 / 0.201 | 0.239 / 0.256 | 0.177 / 0.174 | 0.757 / 0.775 |
| EGAAC | 86.86 / 87.45 | 31.97 / 36.38 | 90.22 / 90.84 | 0.167 / 0.212 | 0.221 / 0.265 | 0.171 / 0.184 | 0.776 / 0.764 |

Note: the result for 10-fold cross-validation and independent dataset are seperated by the symbol "/".

**Table S5. Performance comparison of different encoding schemes on human_G dataset based on 10-fold cross-validation and independent test.**

| **Encodings** | **Acc(%)** | **Sn(%)** | **Sp(%)** | **MCC** | **F1-Score** | **AUPRC** | **AUROC** |
| --- | --- | --- | --- | --- | --- | --- | --- |
| CKSAAP | 87.15 / 87.37 | 30.55 / 29.05 | 90.09 / 90.31 | 0.143 / 0.134 | 0.19 / 0.181 | 0.125 / 0.115 | 0.633 / 0.624 |
| One-hot | 87.51 / 87.72 | 37.0 / 38.51 | 90.13 / 90.2 | 0.186 / 0.195 | 0.226 / 0.231 | 0.177 / 0.184 | 0.73 / 0.738 |
| BLOSUM62 | 87.46 / 87.59 | 37.76 / 38.3 | 90.04 / 90.08 | 0.19 / 0.192 | 0.229 / 0.229 | 0.181 / 0.185 | 0.732 / 0.738 |
| Z-scales | 87.6 / 87.39 | 40.92 / 39.73 | 90.02 / 89.79 | 0.21 / 0.197 | 0.246 / 0.232 | 0.194 / 0.189 | 0.744 / 0.748 |
| AAF | 87.63 / 87.65 | 41.04 / 40.09 | 90.05 / 90.05 | 0.211 / 0.203 | 0.247 / 0.238 | 0.195 / 0.19 | 0.747 / 0.745 |
| EGAAC | 87.61 / 87.73 | 41.16 / 40.6 | 90.02 / 90.11 | 0.212 / 0.207 | 0.247 / 0.241 | 0.195 / 0.192 | 0.773 / 0.774 |
| EAAC | 87.73 / 87.92 | 43.66 / 43.38 | 90.02 / 90.17 | 0.228 / 0.225 | 0.26 / 0.256 | 0.205 / 0.202 | 0.787 / 0.787 |
| Aaindex | 87.89 / 88.16 | 46.35 / 46.48 | 90.05 / 90.26 | 0.245 / 0.246 | 0.274 / 0.274 | 0.218 / 0.222 | 0.787 / 0.797 |

Note: the result for 10-fold cross-validation and independent dataset are seperated by the symbol "/".

**Table S6. Performance comparison of different encoding schemes on mouse_L dataset based on 10-fold cross-validation and independent test.**

| **Encodings** | **Acc(%)** | **Sn(%)** | **Sp(%)** | **MCC** | **F1-Score** | **AUPRC** | **AUROC** |
| --- | --- | --- | --- | --- | --- | --- | --- |
| CKSAAP | 81.43 / 81.24 | 33.05 / 33.28 | 90.07 / 89.91 | 0.243 / 0.243 | 0.35 / 0.352 | 0.286 / 0.284 | 0.679 / 0.675 |
| One-hot | 80.65 / 80.68 | 23.09 / 23.16 | 90.93 / 91.07 | 0.159 / 0.163 | 0.266 / 0.268 | 0.268 / 0.267 | 0.681 / 0.68 |
| Z-scales | 80.52 / 80.38 | 24.94 / 24.69 | 90.45 / 90.45 | 0.171 / 0.168 | 0.28 / 0.278 | 0.276 / 0.275 | 0.7 / 0.694 |
| BLOSUM62 | 80.45 / 80.57 | 25.99 / 26.08 | 90.19 / 90.42 | 0.177 / 0.182 | 0.287 / 0.291 | 0.283 / 0.285 | 0.7 / 0.698 |
| AAF | 80.69 / 80.77 | 24.47 / 24.63 | 90.73 / 90.91 | 0.171 / 0.176 | 0.278 / 0.282 | 0.28 / 0.282 | 0.702 / 0.702 |
| EGAAC | 81.17 / 81.03 | 31.58 / 31.57 | 90.03 / 89.97 | 0.229 / 0.228 | 0.337 / 0.337 | 0.324 / 0.324 | 0.758 / 0.754 |
| Aaindex | 81.09 / 81.0 | 31.15 / 31.08 | 90.01 / 90.02 | 0.224 / 0.224 | 0.333 / 0.334 | 0.328 / 0.327 | 0.77 / 0.769 |
| EAAC | 82.02 / 82.03 | 34.8 / 35.07 | 90.46 / 90.51 | 0.266 / 0.27 | 0.37 / 0.374 | 0.362 / 0.365 | 0.786 / 0.785 |

Note: the result for 10-fold cross-validation and independent dataset are seperated by the symbol "/".

**Table S7. Performance comparison of different encoding schemes on rice_L dataset based on 10-fold cross-validation and independent test.**

| **Encodings** | **Acc(%)** | **Sn(%)** | **Sp(%)** | **MCC** | **F1-Score** | **AUPRC** | **AUROC** |
| --- | --- | --- | --- | --- | --- | --- | --- |
| CKSAAP | 83.24 / 83.42 | 23.25 / 24.37 | 90.12 / 90.2 | 0.129 / 0.14 | 0.222 / 0.232 | 0.159 / 0.166 | 0.595 / 0.6 |
| One-hot | 85.41 / 85.29 | 43.76 / 40.4 | 90.19 / 90.44 | 0.304 / 0.281 | 0.381 / 0.361 | 0.327 / 0.306 | 0.758 / 0.739 |
| BLOSUM62 | 85.61 / 85.28 | 47.03 / 42.66 | 90.04 / 90.17 | 0.327 / 0.294 | 0.402 / 0.373 | 0.349 / 0.331 | 0.764 / 0.755 |
| AAF | 85.7 / 85.39 | 47.36 / 44.26 | 90.1 / 90.11 | 0.33 / 0.306 | 0.405 / 0.384 | 0.352 / 0.334 | 0.766 / 0.76 |
| Z-scales | 86.06 / 85.86 | 47.97 / 42.46 | 90.44 / 90.84 | 0.341 / 0.305 | 0.414 / 0.382 | 0.364 / 0.339 | 0.768 / 0.755 |
| Aaindex | 85.94 / 85.74 | 49.79 / 46.49 | 90.09 / 90.24 | 0.349 / 0.326 | 0.421 / 0.401 | 0.381 / 0.379 | 0.79 / 0.788 |
| EGAAC | 85.66 / 85.43 | 47.56 / 47.36 | 90.04 / 89.8 | 0.331 / 0.325 | 0.405 / 0.401 | 0.374 / 0.356 | 0.795 / 0.785 |
| EAAC | 86.24 / 86.13 | 49.78 / 49.4 | 90.44 / 90.34 | 0.355 / 0.351 | 0.426 / 0.423 | 0.397 / 0.378 | 0.808 / 0.804 |

Note: the result for 10-fold cross-validation and independent dataset are seperated by the symbol "/".

**Table S8. Performance comparison of different encoding schemes on rice_S dataset based on 10-fold cross-validation and independent test.**

| **Encodings** | **Acc(%)** | **Sn(%)** | **Sp(%)** | **MCC** | **F1-Score** | **AUPRC** | **AUROC** |
| --- | --- | --- | --- | --- | --- | --- | --- |
| CKSAAP | 82.18 / 81.74 | 27.89 / 28.23 | 90.19 / 89.58 | 0.185 / 0.179 | 0.287 / 0.283 | 0.215 / 0.205 | 0.646 / 0.637 |
| One-hot | 81.34 / 81.05 | 20.8 / 21.06 | 90.27 / 89.85 | 0.118 / 0.114 | 0.223 / 0.221 | 0.209 / 0.207 | 0.656 / 0.662 |
| BLOSUM62 | 81.27 / 81.29 | 21.97 / 21.11 | 90.02 / 90.12 | 0.126 / 0.118 | 0.232 / 0.224 | 0.216 / 0.21 | 0.666 / 0.673 |
| AAF | 81.45 / 81.4 | 22.09 / 23.03 | 90.21 / 89.96 | 0.13 / 0.135 | 0.234 / 0.24 | 0.217 / 0.218 | 0.669 / 0.673 |
| Z-scales | 81.87 / 81.57 | 20.72 / 20.69 | 90.89 / 90.5 | 0.126 / 0.12 | 0.227 / 0.223 | 0.218 / 0.212 | 0.67 / 0.676 |
| Aaindex | 82.27 / 82.5 | 26.08 / 24.67 | 90.56 / 90.98 | 0.174 / 0.167 | 0.274 / 0.265 | 0.252 / 0.244 | 0.724 / 0.724 |
| EGAAC | 82.45 / 82.72 | 27.75 / 28.57 | 90.53 / 90.66 | 0.19 / 0.199 | 0.289 / 0.297 | 0.265 / 0.276 | 0.734 / 0.746 |
| EAAC | 82.42 / 82.7 | 30.72 / 31.55 | 90.05 / 90.2 | 0.209 / 0.219 | 0.31 / 0.318 | 0.28 / 0.286 | 0.74 / 0.753 |

Note: the result for 10-fold cross-validation and independent dataset are seperated by the symbol "/".

**Table S9. Performance comparison of different encoding schemes on rice_F dataset based on 10-fold cross-validation and independent test.**

| **Encodings** | **Acc(%)** | **Sn(%)** | **Sp(%)** | **MCC** | **F1-Score** | **AUPRC** | **AUROC** |
| --- | --- | --- | --- | --- | --- | --- | --- |
| One-hot | 82.96 / 82.37 | 19.12 / 17.15 | 90.68 / 90.54 | 0.1 / 0.08 | 0.194 / 0.178 | 0.171 / 0.162 | 0.617 / 0.598 |
| CKSAAP | 83.62 / 83.2 | 30.7 / 30.28 | 90.04 / 89.84 | 0.197 / 0.192 | 0.288 / 0.287 | 0.201 / 0.196 | 0.629 / 0.645 |
| Z-scales | 83.06 / 82.6 | 19.74 / 17.41 | 90.74 / 90.77 | 0.106 / 0.085 | 0.201 / 0.182 | 0.177 / 0.163 | 0.634 / 0.592 |
| BLOSUM62 | 83.11 / 81.7 | 19.03 / 16.02 | 90.86 / 89.94 | 0.102 / 0.061 | 0.195 / 0.163 | 0.178 / 0.157 | 0.635 / 0.588 |
| AAF | 83.28 / 82.81 | 18.24 / 15.73 | 91.17 / 91.22 | 0.099 / 0.075 | 0.191 / 0.169 | 0.175 / 0.164 | 0.639 / 0.612 |
| Aaindex | 82.74 / 81.76 | 22.62 / 20.15 | 90.02 / 89.49 | 0.123 / 0.095 | 0.22 / 0.198 | 0.194 / 0.18 | 0.678 / 0.641 |
| EGAAC | 83.08 / 82.77 | 22.55 / 23.26 | 90.42 / 90.23 | 0.129 / 0.134 | 0.223 / 0.231 | 0.205 / 0.206 | 0.707 / 0.684 |
| EAAC | 83.47 / 83.26 | 29.26 / 27.99 | 90.04 / 90.19 | 0.184 / 0.177 | 0.277 / 0.271 | 0.234 / 0.213 | 0.731 / 0.706 |

Note: the result for 10-fold cross-validation and independent dataset are seperated by the symbol "/".

**Table S10. Performance comparison of different encoding schemes on rice_G dataset based on 10-fold cross-validation and independent test.**

| **Encodings** | **Acc(%)** | **Sn(%)** | **Sp(%)** | **MCC** | **F1-Score** | **AUPRC** | **AUROC** |
| --- | --- | --- | --- | --- | --- | --- | --- |
| CKSAAP | 82.77 / 82.36 | 28.26 / 28.76 | 90.02 / 89.5 | 0.18 / 0.177 | 0.278 / 0.277 | 0.214 / 0.216 | 0.634 / 0.631 |
| One-hot | 82.8 / 82.65 | 28.07 / 27.8 | 90.07 / 89.96 | 0.179 / 0.175 | 0.277 / 0.274 | 0.233 / 0.239 | 0.658 / 0.655 |
| BLOSUM62 | 82.97 / 82.64 | 29.23 / 28.56 | 90.12 / 89.85 | 0.191 / 0.18 | 0.287 / 0.279 | 0.236 / 0.242 | 0.661 / 0.653 |
| AAF | 82.97 / 82.98 | 29.58 / 29.58 | 90.06 / 90.1 | 0.193 / 0.194 | 0.289 / 0.29 | 0.239 / 0.249 | 0.668 / 0.665 |
| Z-scales | 82.9 / 82.96 | 29.22 / 30.25 | 90.03 / 89.99 | 0.189 / 0.198 | 0.286 / 0.295 | 0.24 / 0.25 | 0.668 / 0.665 |
| EGAAC | 83.2 / 83.57 | 31.31 / 33.32 | 90.1 / 90.26 | 0.209 / 0.23 | 0.304 / 0.323 | 0.26 / 0.282 | 0.702 / 0.708 |
| Aaindex | 83.22 / 83.07 | 32.13 / 31.86 | 90.01 / 89.9 | 0.215 / 0.211 | 0.31 / 0.307 | 0.262 / 0.27 | 0.71 / 0.706 |
| EAAC | 83.42 / 83.49 | 33.3 / 34.32 | 90.09 / 90.05 | 0.226 / 0.235 | 0.32 / 0.328 | 0.268 / 0.284 | 0.714 / 0.716 |

Note: the result for 10-fold cross-validation and independent dataset are seperated by the symbol "/".

**Table S11. Performance comparison based on the independent dataset. The models were trained with the dataset of 30% and 40% sequence identity,**

**respectively.**

| **Datasets** | **30% similarity** | | | | | | | **40% similarity** | | | | | | |
| --- | --- | --- | --- | --- | --- | --- | --- | --- | --- | --- | --- | --- | --- | --- |
|  | **Acc (%)** | **Sn (%)** | **Sp (%)** | **MCC** | **F1-Score** | **AUPRC** | **AUROC** | **Acc (%)** | **Sn (%)** | **Sp (%)** | **MCC** | **F1-Score** | **AUPRC** | **AUROC** |
| Human_L | 88.96 | 65.51 | 90.05 | 0.348 | 0.346 | 0.331 | 0.895 | 88.93 | 66.32 | 90.06 | 0.361 | 0.363 | 0.347 | 0.901 |
| Human_U | 88.55 | 59.37 | 90.03 | 0.322 | 0.334 | 0.296 | 0.885 | 88.55 | 60.34 | 90.01 | 0.33 | 0.342 | 0.304 | 0.891 |
| Human_O | 88.81 | 46.23 | 90.29 | 0.211 | 0.217 | 0.152 | 0.843 | 88.54 | 47.75 | 90.04 | 0.221 | 0.228 | 0.148 | 0.865 |
| Human_K | 88.06 | 56.78 | 90.03 | 0.331 | 0.358 | 0.329 | 0.879 | 87.65 | 51.23 | 90.07 | 0.303 | 0.341 | 0.317 | 0.851 |
| Human_G | 88.24 | 49.46 | 90.09 | 0.257 | 0.277 | 0.212 | 0.855 | 88.07 | 48.03 | 90.08 | 0.263 | 0.275 | 0.232 | 0.868 |
| Mouse_L | 83.60 | 46.98 | 90.06 | 0.366 | 0.461 | 0.449 | 0.846 | 83.42 | 46.97 | 90.01 | 0.367 | 0.462 | 0.456 | 0.851 |
| Rice_L | 87.12 | 60.99 | 90.05 | 0.430 | 0.488 | 0.490 | 0.875 | 86.56 | 56.54 | 90.00 | 0.399 | 0.464 | 0.453 | 0.859 |
| Rice_S | 84.10 | 43.08 | 90.07 | 0.317 | 0.407 | 0.360 | 0.816 | 84.25 | 44.94 | 90.02 | 0.332 | 0.421 | 0.379 | 0.832 |
| Rice_F | 84.38 | 42.27 | 90.07 | 0.304 | 0.390 | 0.347 | 0.822 | 83.97 | 35.56 | 90.04 | 0.241 | 0.330 | 0.297 | 0.807 |
| Rice_G | 84.08 | 39.68 | 90.01 | 0.281 | 0.370 | 0.323 | 0.799 | 84.19 | 40.49 | 90.02 | 0.287 | 0.375 | 0.338 | 0.809 |
