# Supplementary material for: ResNetKhib: a novel cell type-specific tool for predicting lysine 2-hydroxyisobutylation sites via transfer learning
Source: Brief Bioinform. 2023 Mar 4;24(2):bbad063. doi: 10.1093/bib/bbad063 (PMC10185920; doi:10.1093/bib/bbad063)
Supplement: Response_letter_Final_bbad063 [file response_letter_final_bbad063.docx]

Dear Prof Bishop,

We submit the revised version of our manuscript BIB-22-2039.R1. We thank you and the reviewers for your time and effort in providing constructive suggestions that undoubtedly have helped us improve the manuscript’s presentation.

We have carefully addressed the reviewers’ comments and accordingly revised our manuscript. The revisions are highlighted in red font in the revised version of the manuscript, including the enclosed user manual. We also provide detailed point-by-point responses to the reviewers' comments to further ease the adjudication of the revision.

Thank you very much for your consideration. We look forward to hearing from you soon.

With our best regards,

Gensheng Dou, Zhen Chen, and Jiangning Song

**Reviewer: 1**

Comments to the Author

This manuscript proposed a novel bioinformatics tool, ResNetKhib, to predict lysine 2-hydroxyisobutylation sites. The primary workflow of this method is logical, and the webserver is well-designed. I have several comments for the manuscript that need to be fixed.

1. Why did the authors use 40% instead of 30% as the threshold to remove the redundant sequences in the training dataset? 30% is more rigorous and utilised by more research.

**Response:** We appreciate this comment and fully agree that 30% is more rigorous than 40% as the sequence identity threshold to remove the redundant sequences in the training dataset. Five tools have been developed to predict Khib sites. DeepKhib, iLys-Khib and Ju *et al* ‘s method. used 40% as the threshold, while KhibPred used 30% as the threshold. For 2-hydr_Ensemble, we can not find the sequence identity information. In order to enable the comparison with DeepKhib, which is the only currently available tool for Khib site prediction, we also used the 40% sequence identity in our study. In the revised manuscript, according to the reviewer’s suggestion, to further assess the robustness of ResNetKhib, we have rebuilt the training dataset with the 30% sequence identity, and retrained the models. The performance of seven retrained models decreased slightly, while the performance of three retrained models improved compared with the models trained on the datasets with the 40% sequence identity. Please refer to the **Supplementary Table S11** for more details. We have also discussed the results in the “**Evaluation of the prediction models on different datasets**” section on Page 18 of the revised manuscript.

2. The authors are suggested to explain why they used these feature encoding schemes to represent the sequences. For example, why not only use sequence embeddings or one-hot encodings, which are simple and more popular for deep learning algorithms?

**Response:** We appreciate this comment. We used these feature encoding schemes to represent the sequences due to the following considerations: To develop an effective machine learning method for Khib site prediction, multi-encodings and machine learning algorithms need to be evaluated. Thus, we collected eight commonly used feature encoding schemes and sequence embedding method to represent the sequence, and used both conventional machine learning-based and deep learning-based algorithms to integrate these encodings. The random forest algorithm was used to integrate the commonly used eight types encodings, while the one-dimensional convolution-based ResNet was used to integrate the one-hot encoding and embeddings. The results showed that the ResNet-based models performed better than the RF-based models. The ResNet model with embedding and one-hot encoding were named ResNetKhib_WE_ and ResNetKhib_one-hot_, respectively. Considering that ResNetKhib_WE_ performed better than ResNetKhib_one-hot_, we chose the ResNetKhib_WE_ models as the final prediction models of ResNetKhib. We have accordingly provided a detailed explanation in the “*Feature encoding schemes employed*” section on Page 9 of the revised manuscript.

3. It would be better to visualise the features learned inside the deep learning framework of ResNetKhib.

**Response:** We appreciate this comment. Following the reviewer’s suggestion, we have accordingly employed the t-SNE algorithm to plot and visualize the sample distributions using the features of the embedding layer, the last convolutional layer and the fully connected layer inside the deep learning framework ResNetKhib based on the independent dataset. The results are shown in the **Supplementary Figure S4**. We can see that in the embedding layer, the positive and negative samples were mixed together. However, these two types of samples were clearly separated from each other after the convolutional layer and further separated after the fully connected layer. These indicate that the deep learning framework of ResNetKhib could effectively learn the feature representations and thereby distinguish the positive from negative samples. We have accordingly discussed these results by adding a feature visualization paragraph in the “*Performance evaluation on 10-fold cross-validation and independent tests*” section on Page 17 of the revised manuscript.

4. The precision-recall curve should also be considered to evaluate the model performance, as the dataset is highly imbalanced.

**Response:** Thanks for the comment. We have accordingly added the precision-recall curves in the **Supplementary Figure S5**.

5. The loss-accuracy curve of training should be provided.

**Response:** Thanks for the comment. We have accordingly added the loss-accuracy curves of the model training process in the **Supplementary Figure S2** of the revised manuscript.

6. How did the author avoid overfitting during model training?

**Response:** We appreciate this comment. In this study, we used the dropout to avoid overfitting during model training. In ResNetKhib, we added a dropout operation after each convolution layer, with the dropout rate set as 0.5. The minor performance difference between 10-fold cross-validation and independent test illustrated that the dropout operation can effectively avoid overfitting. We have added a detailed description of this strategy in the “*Model training strategy*” section on Page 13 of the revised manuscript.

**Reviewer: 2**

Comments to the Author

In this manuscript, authors first found that Khib sites may have distinct characteristics for different cell types of the same species. They, they proposed deep learning-based approach named ResNetKhib, which leverages both the one-dimensional convolution and transfer learning to enable and improve the prediction of cell type-specific Khib sites. This work is comprehensive and worthy of publication. I have the following comments below for authors to consider improving the quality of this work:

1. In the “EGAAC encoding” section, the 20 amino acids are categorized in to five major groups according to their physicochemical properties. However, we do not know what these properties are? It would be better if the author could provide the information.

**Response:** We appreciate this comment. We have accordingly added detailed information about the physicochemical properties of five major amino acid groups in the “*EGAAC encoding*” section on Page 10 of the revised manuscript. Please refer to the corresponding sections for details.

2. In the “Model training strategy” section, the author trained the model based on the binary cross-entropy loss function. What is the definition of binary cross-entropy loss function? It is better to list the formula of binary cross-entropy function in the manuscript.

**Response:** We appreciate this comment. We have accordingly added the definition of the binary cross-entropy loss function in the “*Model training strategy*” on Page 13 of the revised manuscript. Please refer to the equation 2 for details.

3. The computational efficiency of the developed deep learning method should be described – how long will it take the online web server to complete the prediction of a typical protein sequence with the average sequence length (e.g. 500 AA)?

**Response:** We appreciate this comment. In general, for a typical protein sequence with around 500 amino acid residues, completing a single prediction task would take about five seconds. We have accordingly added a description of the computational efficiency of the web server in the “*Implementation of the ResNetKhib web server*” section on Page 20 of the revised manuscript. Please refer to the corresponding section for details.

4. For the result page, the author provided a table to list the prediction result and an interactive graphical to view the statistical summary of the prediction result. However, the author should also provide the threshold value interval, which may make it easier for users to understand the results.

**Response:** Thanks for the comment. We have accordingly added the threshold value interval on the result page of the web server. Please refer to the web server and the updated **Figure 6** of the revised manuscript.

**Reviewer: 3**

Comments to the Author

Authors developed a deep learning-based approach, termed ResNetKhib, which leverages both the one-dimensional convolution and transfer learning to enable and improve the prediction of cell type-specific 2-hydroxyisobutylation sites. ResNetKhib can predict Khib sites for four human cell types, mouse liver cell and three rice cell types. This work is meaningful in this field.

**Response:** Thanks for your positive comments.

1. Authors should give a detailed demonstration of the outline of this work.

**Response:** We appreciate your comment. We have added a section named “*Outline of the work*” at the beginning of the “*Materials and Methods*” section. Please refer to the corresponding section for details.

2. In order to show the performances of this work, authors should be utilized the F1 score in this manuscript.

**Response:** We appreciate your comment. We have accordingly added and used the F1-score in the revised manuscript. Please refer to the “*Performance evaluation strategies*”, “*Performance evaluation on 10-fold cross-validation and independent tests*” sections and updated **Table 4** for details.

3. In this work, authors utilized the transfer learning to deal with the 2-hydroxyisobutylation sites of lysine. It was suggested that authors should show how to utilize the transfer learning.

**Response:** We appreciate your comment. In this study, the models for two general datasets (*i.e.*, Human_G and rice_G) were trained and optimized based on the binary cross-entropy (BCE) loss function using the Adam algorithm. The maximum training cycles were set as 1000 epochs to ensure that the loss function value converged. The early-stopping strategy was also used during the model training process. We applied the transfer learning strategy to train cell type-specific models in humans and rice. Taking the training process of the Human_O model as an example, the well-trained Human_G model was used as the pre-trained model. We first loaded the parameters of the pre-trained model to the ResNetKhib network and then implemented the fine-tuning technique with no parameters constrained. The transfer learning process was also trained and optimized based on the BCE loss function using the Adam algorithm, with the learning rate set to 0.01. The maximum training cycles were set as 500 epochs, and the early stopping strategy was employed to avoid overfitting. The loss-accuracy curves on each dataset are shown in the **Supplementary Figure S2**. We have accordingly added a new section “*Transfer learning for cell type-specific models*” to describe how to utilize the transfer learning in the revised manuscript.

4. Some grarmmar and spelling mistakes should be avoided in this work.

**Response:** We appreciate your comment. We have accordingly checked and revised the grammar and spelling mistakes in the revised manuscript.

5. Some references, 10.1016/j.chemolab.2021.104351 and 10.1155/2022/9470683, should be discussed in this work.

**Response:** We appreciate your comment. We have accordingly discussed these two works in the revised manuscript. Please refer to the fourth paragraph of the “*Introduction*” and “*Performance evaluation strategies*” sections of the revised manuscript.

**Reviewer: 4**

Comments to the Author

The authors have developed a deep learning-based approach, termed “ResNetKhib”, to improve the prediction of cell type-specific lysine 2-hydroxyisobutylation (Khib) sites. This tool leverages the one-dimensional convolution for Khib sites and can predict Khib sites in different cell types, including human, mouse liver, and rice. Comparing different parameters of statistical approaches between training and testing dataset, this tool achieves a better performance, with higher AUC, than RF-based predictors and other currently available Khib site prediction tools. Many parts look good, however, many issues and concerns about the usefulness and application of the model should be clarified or need to have further revision or discussion.

1. Many modifications present on lysine residue, including acetylation and 2-hydroxyisobutylation (Khib). Both of the modifications also cause and regulate by the same enzymes, such as EP300, HDAC2 and HDAC3. It means the modifications may be generated by the same mechanism or enzymatic recognition sites/motifs. I am curious about how the authors clarify the modified lysine can specifically conjugate 2-hydroxyisobutylateion but not acetylation? Do the authors exclude all of modified lysines? Or, in case, if the “positive” samples of Khib sites can compare to other lysine modification sites (especially the acetylation, suggesting at least three modifications dataset), it will be more significant for site-specific and cell-type-specific prediction.

**Response:** We appreciate your comment. In addition to protein acetyltransferase, EP300 can use different acyl-CoA substrates, such as (2E)-butenoyl-CoA (crotonyl-CoA), butanoyl-CoA (butyryl-CoA), 2-hydroxyisobutanoyl-CoA (2-hydroxyisobutyryl-CoA), lactoyl-CoA or propanoyl-CoA (propionyl-CoA), and is able to mediate protein crotonylation, butyrylation, 2-hydroxyisobutyrylation, lactylation or propionylation, respectively [1-4]. While HDAC2 and HDAC3 also function as the protein-lysine deacylase by recognizing other acyl groups: catalyze removal of (2E)-butenoyl (crotonyl) and 2-hydroxyisobutanoyl (2-hydroxyisobutyryl) acyl groups from lysine residues, leading to protein decrotonylation and de-2-hydroxyisobutyrylation, respectively [5-7]. It seems that all of these modifications are regulated by the same enzymes, and Khib sites may have some overlap with other acylations such as succinylation, acetylation and crotonylation et al. However, the molecular mechanism of each acylation process is different. There is an internal regulatory element for EP300 to select substrates for different acylation [3]. For example, Huang et al. reported that EP300 differentially regulates the Khib and Kac on distinct lysine sites, with only 6 out of the 149 EP300-targeted Khib sites overlapping with the 693 EP300-targeted Kac sites, and demonstrated that diverse cellular proteins, particularly glycolytic enzymes, are targeted by EP300 for Khib but not for Kac [3]. Feng et al. also performed a systematic identification of lysine Khib sites to uncover the critical role of Khib in wheat biological regulations and found only 4 overlap modification sites for Khib, Kac and Ksucc in 3684 modification sites (see **Figure** 1 below), thereby demonstrating a unique regulatory mechanism for EP300 to differentially target these acylations.

**Figure 1. Venn diagrams showing the overlap of Kac, Ksucc and Khib sites [8].**

In this study, our major focus was on predicting Khib sites from non-Khib sites. A lysine residue is experimentally verified to be 2-hydroxyisobutyrylated was considered as a positive sample, irrespective of whether it can be acetylated or crotonylated. Considering the fact that a lysine residue may be modified by one more acylations, to address this, the dataset for all types of acylation should be collected, labeled, analyzed, and train a multi-task model to predict how many types of acylation modification can occur on the same lysine residue simultaneously. We plan to investigate this perspective in the future work.

2. The strategies are similar to lots of studies for one-dimensional modification site prediction. Therefore, the dataset collection and filtering is important for the analysis to achieve the prediction accuracy. In the result of two sample logo from different cell types, some motifs are similar in all of specimen (human/mouse/rice). I suggest the authors should further process and clean the datasets to remove interference and generate the “cell-types-specific motifs”.

**Response:** We appreciate your comment. To further explore the cell type-specific motifs, we have compared the distributions and preferences of the flanking residues between the positive samples of any two different cell types in a species (**Supplementary Figure S3**). For the Human_L dataset, the amino acids K and R are predominantly significantly enriched at +1 position compared with Human_K, Human_O and Human_U datasets (**Supplementary Figures S3A, S3B, and S3C**). For the Rice_L dataset, K and R are enriched at +1 position compared with Rice_F and Rice_S datasets (**Supplementary Figures S3G and S3I**), while K and R were are over-presented at –1 position for the Rice_F dataset compared with Rice_L and Rice_S dataset (**Supplementary Figures S3G and S3H**). The difference can be observed between any of the two positive datasets. However, there were no unified motifs can be observed for the remaining datasets (expect for the Human_L, Rice_F and Rice_L datasets) when compared with other cell types. We have accordingly added a paragraph to discuss the cell type-specific motifs. Please refer to the third paragraph of the “*Motif conservation analysis of Khib sites in different species and cell types*” section on Page 16 of the revised manuscript.

3. I suggest the authors should provide some real cases for cell types-specific Khib sites in the main text. In addition, the author should also present some cases for real case from ResNetKhib prediction.

**Response:** We appreciate your comment. Following the reviewer’s suggestion, we have plotted the Venn diagrams and analyzed the overlap among different cell types of the same species at the protein and residue levels. As shown in **Supplementary Figure S6**, only 51 K_hib_ sites overlapped in all the four human cell types (**Supplementary Figure S6A**). In contrast, there were 170 K_hib_ sites appearing in all the three rice cell types (**Supplementary Figure S6C**). Interestingly, the number of overlapped 2-hydroxyisobutyrylated proteins was larger than that of overlapped K_hib_ sites in the four human cell types (**Supplementary Figures S6A and S6B**), indicating that the majority of the K_hib_ sites are different in number in the same protein of different human cell types. Taking the EIF3A protein (UniProt ID: Q14152) as an example, seven lysine residues of EIF3A can be 2-hydroxyisobutyrylated. The lysines K100 and K694 were 2-hydroxyisobutyrylated in the kidney cell, while K23, K742, and K775 were 2-hydroxyisobutyrylated in the lung cell. No modified K_hib_ sites were detected in squamous cell carcinoma cells, while K538 and K711 were found to be 2-hydroxyisobutyrylated in HeLa cells. For rice, the rice_S dataset has the largest number of 2-hydroxyisobutyrylated proteins, followed by rice_L and rice_F (**Supplementary Figure S6D**).

In order to test the predictive capability of ResNetKhib, we applied its model to predict the cell type-specific K_hib_ sites in rice. We randomly selected 100 protein sequences from the rice (*Oryza sativa subsp. japonica*) proteome, whose sequences did not overlap with those in the training dataset. We submitted these sequences to the ResNetKhib web server with the “HIGH” confidence. Amongst these 100 protein sequences, 50, 57, and 52 protein sequences were predicted to be 2-hydroxyisobutyrylated in rice flower, leaves, and seeding cells, respectively. Only seven sites and 31 proteins were predicted to be 2-hydroxyisobutyrylated in all the three cell types (**Supplementary Figure S6E and S6F**). Taking the CDKE-1 protein (UniProt ID: Q336M2) as an example, the K59 residue was predicted to be 2-hydroxyisobutyrylated in rice flower cells, while K59 and K259 were predicted to be 2-hydroxyisobutyrylated in leaves. There were no residues predicted to be 2-hydroxyisobutyrylated in the seeding cell. The results suggest that ResNetKhib can be exploited as a useful tool for predicting putative K_hib_ modified proteins and K_hib_ sites in the cell type-specific manner.

We have accordingly added a paragraph to discuss the cell type-specific modified protein and residues in the revised manuscript. Please refer to the third paragraph of the “*Overlap of cell type-specific Khib modified proteins and Khib sites*” section.

4. For the website, the example protein is not from human, but it still can predict the Khib site in human cells? Please check the search/prediction function in the web.

**Response:** We appreciate your comment. It is notworthy that the ResNetKhib web server accepts protein sequences in the FASTA format from any species. It cannot make a judgment about the source of the sequence. For example, if you submit a rice sequence to the human model, ResNetKhib will still generate the prediction result for this sequence. However, such prediction would not be biologically relevant. To ensure the reliability and relevance of the prediction result, we recommend users select the model of the corresponding species or cell type to which their sequence belong when submitting the task.

**References**

1.	Chen Y, Sprung R, Tang Y et al. Lysine propionylation and butyrylation are novel post-translational modifications in histones, Mol Cell Proteomics 2007;6:812-819.

2.	Sabari BR, Tang Z, Huang H et al. Intracellular crotonyl-CoA stimulates transcription through p300-catalyzed histone crotonylation, Mol Cell 2015;58:203-215.

3.	Huang H, Tang S, Ji M et al. p300-Mediated Lysine 2-Hydroxyisobutyrylation Regulates Glycolysis, Mol Cell 2018;70:663-678 e666.

4.	Zhang D, Tang Z, Huang H et al. Metabolic regulation of gene expression by histone lactylation, Nature 2019;574:575-580.

5.	Wei W, Liu X, Chen J et al. Class I histone deacetylases are major histone decrotonylases: evidence for critical and broad function of histone crotonylation in transcription, Cell Res 2017;27:898-915.

6.	Huang H, Luo Z, Qi S et al. Landscape of the regulatory elements for lysine 2-hydroxyisobutyrylation pathway, Cell Res 2018;28:111-125.

7.	Song X, Yang F, Liu X et al. Dynamic crotonylation of EB1 by TIP60 ensures accurate spindle positioning in mitosis, Nat Chem Biol 2021;17:1314-1323.

8.	Feng B, Li S, Wang Z et al. Systematic analysis of lysine 2-hydroxyisobutyrylation posttranslational modification in wheat leaves, PLoS One 2021;16:e0253325.
